# Magnetic Alumina for Chromium Ion Remediation: Isotherm Analysis and Kinetic Modeling

**DOI:** 10.1021/acsomega.6c01792

**Published:** 2026-04-22

**Authors:** Siti Fatimah Md Hanafiah, Northaqifah Hasna Mohamed Khir, Nur Fatien Muhamad Salleh, Nurasmat Mohd Shukri

**Affiliations:** † School of Health Sciences, Universiti Sains Malaysia, Health Campus, 16150 Kubang Kerian, Kelantan, Malaysia; ‡ Faculty of Applied Sciences, Universiti Teknologi MARA, Terengganu Branch, Bukit Besi Campus, 23000 Dungun, Terengganu, Malaysia

## Abstract

Heavy metal contamination in aquatic systems poses serious environmental and health risks, driving the need for high-performance adsorbents with enhanced surface reactivity. This study presents the novel fabrication of cobalt (Co), iron (Fe), and nickel (Ni)-alumina (Al) composites for efficient chromium (Cr) ion remediation. The role of oxygen vacancies (OV), metal substitution, and surface hydroxylation in tuning adsorption efficiency was systematically investigated using physical mixing (PM) and chemical precipitation (CP) methods. Among the composites, FeAl from PM method exhibited the highest Cr adsorption capacity of 500.0 mg g^–1^, achieving 91% removal at pH 7, 40 °C, and an initial concentration of 25 mg L^–1^. Zeta potential analysis confirmed enhanced electrostatic interaction, with FeAl displaying the most negative surface charge of −26.3 mV, which shifted to −15.4 mV after Cr adsorption, indicating effective ion binding and charge neutralization. Comprehensive characterization by FTIR, XRD, SEM–EDX, BET, ESR, and XPS confirmed that Fe incorporation promoted meso–macroporous structure (pore diameter = 296.15 nm), improved magnetic properties, and increased surface hydroxyl density, which collectively facilitated effective Cr ion diffusion and uptake. Adsorption followed the Langmuir model (*R* ^2^ = 0.9899) and pseudo-first-order kinetics, indicating monolayer, predominantly physisorption-driven uptake. Thermodynamic results (Δ*H* = – 26.6 kJ mol^–1^; Δ*S* = – 0.0994 kJ mol^–1^·K^–1^) suggested spontaneous, exothermic adsorption. These findings identify FeAl as a scalable adsorbent with high Cr ion removal efficiency and structural stability, offering potential for sustainable heavy metal remediation in industrial wastewater treatment.

## Graphical abstract

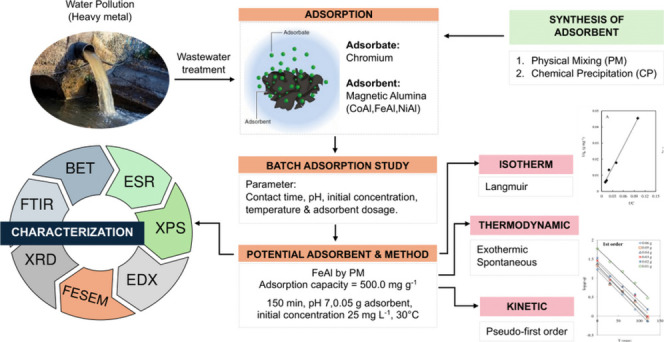

## Introduction

The increasing discharge of heavy metals into the environment, primarily due to industrial activities such as battery manufacturing, fertilizer production, and metal plating, poses a significant threat to both human health and ecosystems. Heavy metals are persistent, nonbiodegradable, and prone to bioaccumulation through the food chain, ultimately reaching humans via ingestion, inhalation, or dermal exposure.^1,2^ Chronic exposure to heavy metals such as Cr, cadmium (Cd), and lead (Pb) has been linked to severe health issues, including renal failure, neurotoxicity, carcinogenicity, and even mortality.^3^ Regulatory agencies such as the Environmental Protection Agency (EPA) and the World Health Organization (WHO) have imposed stringent controls on toxic metals due to their bioavailability and potential to impair aquatic ecosystems, thereby driving the need for cost-effective and scalable remediation technologies.^4^ Among these contaminants, chromium (Cr) is of particular concern due to its water solubility advantage, high mobility and toxicity, in both trivalent (Cr­(III)) and hexavalent (Cr­(VI)) forms. Cr­(VI) is classified as a Group I human carcinogen,^5^ while Cr­(III), although less toxic, can still pose environmental risks at elevated concentrations.^6,7^ Given the toxicity of both Cr­(III) and Cr­(VI), their removal from aqueous environments is of critical importance.^7,8^ Several treatment technologies, including membrane filtration,^9^ separation filtration,^10^ coagulation–flocculation^11^ and adsorption^12^ have been explored for wastewater purification. Among these, adsorption has emerged as a highly efficient and economically viable approach, offering advantages such as low cost, high removal efficiency, operational simplicity, and environmental sustainability. Various adsorbents have been developed for Cr­(III) removal, including activated carbon,^13,14^ zeolites,^14,15^ graphene derivatives,^15,16^ mesoporous silica,^17^ and agricultural waste-based biosorbents such as corncobs,^18^ potato peels^19^ and sugar cane bagasse.^20^ Notably, the modification of biochar and biopolymers has enhanced the adsorption of heavy metals such as cadmium, copper, and lead from industrial effluents.^21,22^ In particular, nanomaterials, such as alumina (Al_2_O_3_), have drawn considerable attention due to their high surface area, porosity, and remarkable adsorption capacity, making them promising candidates for wastewater treatment.^23,24^ Al_2_O_3_ exists in multiple polymorphic forms, including γ, α, δ, θ, and η phases, each with distinct crystallographic structures, surface characteristics, and thermal stability profiles. Several studies have demonstrated the effectiveness of modified α-alumina as an adsorbent for antibiotic removal, with the removal mechanism mainly governed by the electrostatic and hydrophobic interactions between α-alumina surface sites and the antibiotic molecules.^25,26^ These diverse alumina phases underscore the importance of structural control in designing high-performance. Among these, γ-alumina is the most widely used in adsorption applications due to its high surface area, mesoporosity, and abundant surface hydroxyl groups, which are critical for facilitating metal ion interactions and surface complexation mechanisms.^27^ Moreover, γ-Al_2_O_3_ can be synthesized at relatively low temperatures of <600 °C, making it more amenable to functionalization with magnetic and transition metal.^28^ However, conventional alumina-based adsorbents often suffer from low selectivity, difficulty in recovery, and limited adsorption efficiency under varying pH conditions. Recent advances in magnetic oxide-modified materials have shown promise in addressing these limitations by enabling facile recovery and enhanced adsorption efficiency, but the development of composite materials, particularly incorporating magnetic properties with optimized surface functionalization for enhanced heavy metal adsorption, remains underexplored.^29,30^ These limitations underscore the need for novel adsorbents with enhanced capacity, improved stability, and efficient magnetic separation capabilities to facilitate practical applications in wastewater treatment. The novelty of this study lies in the development of magnetic alumina composites through the incorporation of Fe, Co, and Ni via PM and CP methods. Cr removal from aqueous solutions is used as a model system to evaluate the adsorption efficiency. This study systematically investigates how the incorporation of Fe, Co, and Ni in alumina alters interfacial properties, focusing on oxygen vacancies, isomorphous substitution, and surface hydroxylation to enhance Cr adsorption selectivity and kinetics. Comprehensive material characterization was conducted using FTIR, XRD, SEM-EDX, BET, ESR, XPS and zeta potential to elucidate the underlying adsorption mechanisms. Batch adsorption studies were conducted to evaluate the effects of key operational parameters, including contact time, pH, initial Cr concentration, adsorbent dosage, and temperature, on Cr ion adsorption. By bridging nanomaterial surface engineering with adsorption science, this study aims to understand on how surface structural modifications govern adsorbate interactions, while offering a practical and scalable route for heavy metal remediation to develop scalable and efficient technologies for heavy metal remediation.

## Experimental Section

### Materials

Aluminum oxide (Al_2_O_3_), iron­(II) nitrate (Fe­(NO_3_)_2_), cobalt­(II) nitrate (Co (NO_3_)_2_), nickel­(II) nitrate (Ni (NO_3_)_2_), and chromium­(III) nitrate (Cr (NO_3_)_3_) were purchased from Merck. All reagents were of analytical grade and used as received. Adjustments to the pH were made using 1 M hydrochloric acid and 1 M sodium hydroxide. The water solvent used was laboratory-made deionized water.

### Preparation of Adsorbents

Magnetic Al adsorbents were synthesized using three different magnetic compounds; Co, Fe and Ni and two methods of physical mixing (PM) and chemical precipitation (CP). Adsorbents obtained through PM were designated as FeAl, CoAl, and NiAl,^16^ while CP-derived products were labeled FeAl­(C), CoAl­(C), and NiAl­(C).^31,32^ For the PM method, a total of 2 g Al was mixed with 20 mL of deionized water and stirred for 10 min at 40 °C. Simultaneously, 5% (w/w) Fe­(NO_3_)_2_ was added to 35 mL of distilled water, forming a homogeneous solution. The Fe solution was then poured into the Al solution and stirred at 350 rpm for 3 h at 80 °C. The pH was maintained at pH 7 ± 0.2 using 1 M NaOH and 1 M HCl to prevent premature precipitation. The powder form was then aged in an oven at 100 °C for 2 h before being calcined at 550 °C for 3 h. The calcined form of FeAl was then left in a desiccator to remove moisture before being stored in an airtight sample bottle upon adsorption. The procedure was repeated with different magnetic compounds, Co and Ni, resulting in CoAl and NiAl. The second approach of CP began with mixing 2 g Al and 5% (w/w) Fe­(NO_3_)_2_ into 10 mL of methanol for 1 h. The mixture was then stirred at 350 rpm for 30 min with 5 mL of acetic acid. The pH was maintained at pH 7 ± 0.2 using 1 M NaOH and 1 M HCl. The powder form was then aged in an oven at 100 °C for 2 h before being calcined at 550 °C for 3 h. The FeAl­(C) product was then cooled in a desiccator to remove any moisture and stored in an airtight bottle sample after the adsorption process. The procedure was repeated with different magnetic compounds, Co and Ni, resulting in CoAl­(C) and NiAl­(C).

### Characterization

Comprehensive characterization of the synthesized adsorbents was performed using FTIR, SEM, EDX, BET, XRD, XPS and ESR to understand the physicochemical properties. FTIR was conducted using a Bruker Tensor 27 Spectrometer within the range of 400–4000 cm^–1^. The FTIR KBr pellet method was utilized for sample preparation to identify the functional groups of the composites before and after adsorption. SEM and EDX were performed using a FEI QUANTA FEG 450 SEM to examine the surface morphology and elemental composition. The BET method was used to determine specific surface area, pore volume, and pore size distribution, utilizing a Quantachrome AsiQ BET analyzer. XRD analyzed the crystalline structure of the composites on a Bruker D2 Phaser diffractometer over a 2θ range of 20°–90°. XPS was performed using an Axis Ultra DLD XPS, Kratos to determine the chemical oxidation state of the produced adsorbents. ESR analysis was conducted at room temperature using a JEOL JES-FA200 ESR spectrometer to assess the magnetic properties and OV in the composites. Zeta potential measurement Zeta potential (ζ, mV) of the four composites was determined using SurPASS 3 electrokinetic analyzer (Anton Paar,Austria).

### Batch Adsorption System

Serial dilution solutions for Cr ions were prepared from an appropriate 1000 mg/L stock solution. Batch adsorption system was studied to assess the influence factors of different initial pH,^3–11^ adsorbent dosages (0.01–0.06 g), initial concentrations (10–100 mg L^–1^) and temperature (30–50 °C) on the Cr adsorption. 0.02 g of adsorbent was added to a 250 mL beaker containing 150 mL of Cr ion solution at various concentrations. 0.1 M NaOH and 0.1 M HCl were used to adjust the pH value of the Cr aqueous solution. The solution was then stirred at 350 rpm at 30 °C before being collected in centrifuge tubes. Following adsorption, the suspensions underwent centrifugation at 4500 rpm for 5 min, after which the supernatants were filtered through a 0.45 μm filter paper. The residual metal ion concentrations were quantified using Flame Atomic Absorption Spectrophotometer (FAAS), PerkinElmer Analyst 800, operated at a wavelength of 357.87 nm, slit width 0.2 nm, and lamp current 7 mA. An air–acetylene flame was used with an acetylene flow rate of 2.0 L min^–1^ and air flow rate of 13.5 L min^–1^. Blank experiments were conducted to ensure the accuracy and precision of the measurements. Reusability of the adsorbent was evaluated through three consecutive adsorption–desorption cycles. All experiments were performed with triplicate samples, and the results were expressed as removal percentage. The removal percentage (%) was calculated using eq 1.

1 $$\%\;Removal=\left(C_{0}-C_{e}\right)/C_{0}$$

Where *C*_0_ and *C*_e_ are the initial and equilibrium concentrations (mg L^–1^)

### Kinetic, Isotherm and Thermodynamic Study

The isotherm modeling, adsorption kinetics, and thermodynamic studies were conducted to evaluate the adsorption efficiency, mechanism, and governing forces involved in Cr removal using Fe, Co, and Ni-modified alumina composites.^34^

### Kinetic Study

Adsorption kinetics experiments were performed over a contact time range of 30 to 210 min at varying adsorbent dosages (0.02–0.06 g). In adsorption kinetics, adsorption capacity (*q*_e_) plays a crucial role in determining the rate, efficiency, and mechanism of adsorbate uptake over time. eq 2 was used to compute q_e_.

2 $$q_{e}=\left(C_{0}-C_{e}\right)\;V/m$$

Where *m* is the adsorbent weight (g), and *V* is the volume of the reaction system (L).

### Isotherm Study

Adsorption isotherm studies were conducted to investigate the adsorbate–adsorbent interactions at equilibrium. The experimental measures were fitted to the isotherm models; Langmuir, Freundlich, Temkin, and Dubinin–Radushkevich to ascertain the nature of adsorption. The mathematical expressions for Langmuir, Freundlich, Temkin, and Dubinin–Radushkevich isotherms are presented as eq 3 – ([Disp-formula eq6]).

3 $$Langmuir,q_{e}=\frac{q_{m}K_{L}C_{e}}{{1+K}_{L}C_{e}}$$

4 $$Freundlich,q_{e}=K_{F}C_{e}^{1/n_{F}}$$

5 $$Temkin,q_{e}=B\ln\;\ln A_{T}C_{e}$$

6 $$Dubinin-Radushkevich,q_{e}=\left(q_{s}\right)\exp\left(-K_{D}\varepsilon^{2}\right)$$

where *q*_e_ is the amount of metal ions taken up per gram of FeAl (mg g^–1^), *q*_m_ is the maximum adsorption capacity (mg g^–1^), *C*_e_ is the equilibrium concentration of Cr ions (mg L^–1^), n_F_ is a heterogeneity factor, ε is the Polanyi potential, B (RT/bT), b (J mol^–1^) and A (L g^–1^) are the heat of sorption and equilibrium binding constants, *R* denotes the ideal gas constant (8.314 J mol^–^1 K^–1^), and *T* is the absolute temperature (*K*). Meanwhile *K*_L_, *K*_F_, *K*_T_, and *K*_D_ represent the equilibrium constants of the Langmuir (L mg^–1^), Freundlich (mg g^–1^)/(L mg^–1^)^1/n^, Temkin (L mg^–1^) and Dubinin–Radushkevich (mol^2^ (kJ^–2^) models, respectively.

### Thermodynamic Study

To assess the temperature-dependent behavior and feasibility of adsorption, thermodynamic experiments were performed at three distinct temperatures (30, 40, and 50 °C).

## Results and Discussion

### Functional Studies

Figures 1 and 2 presents the FTIR spectra of the synthesized magnetic alumina composites before and after Cr adsorption, providing insights into the functional groups, structural modifications, and interfacial interactions governing the adsorption process.

**Figure 1:**
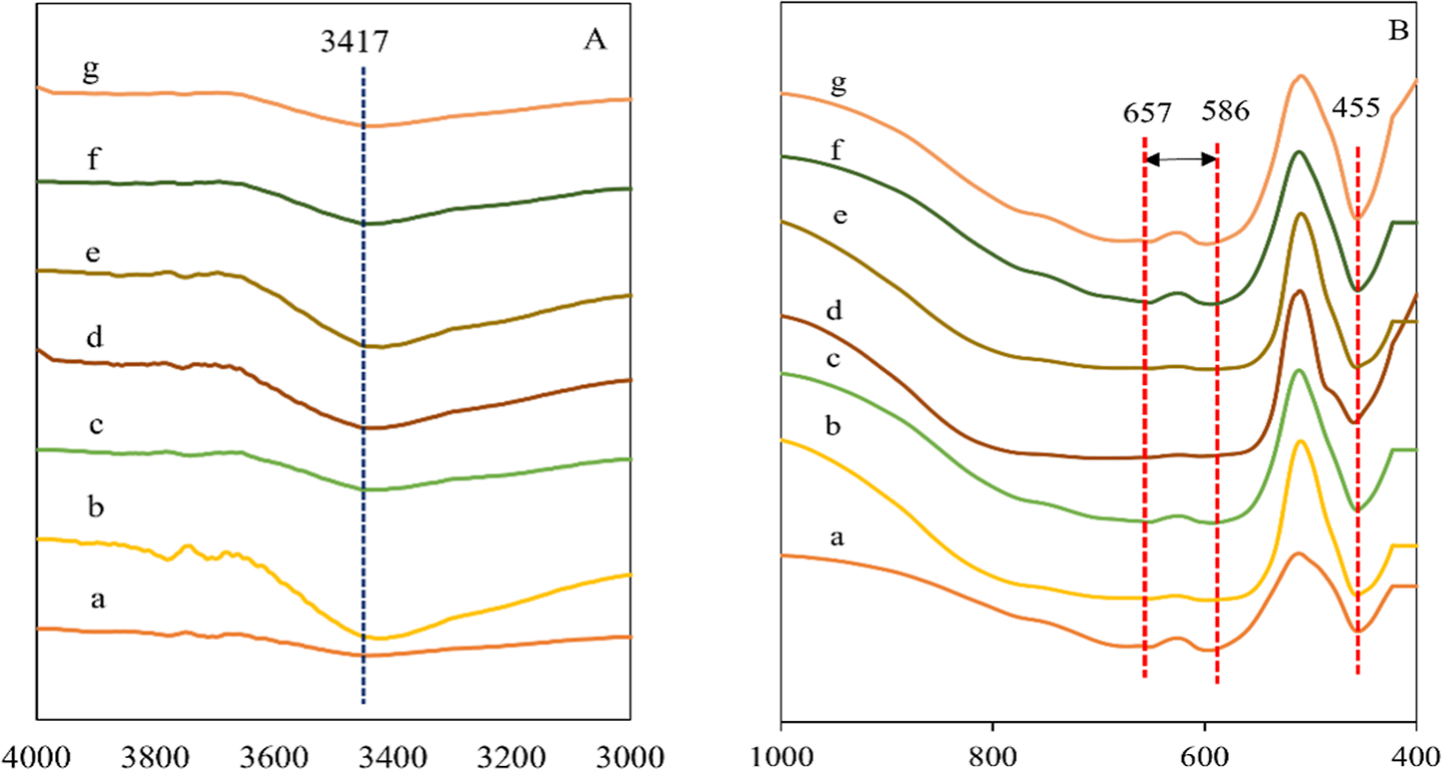
FTIR spectra at range (A) 4000–3000 cm^–1^ and (B) 1000–400 cm^–1^ of adsorbents: (a) Al, (b) FeAl, (c) CoAl, (d) NiAl, (e) FeAl (C), (f) CoAl (C), (g) NiAl (C).

**Figure 2:**
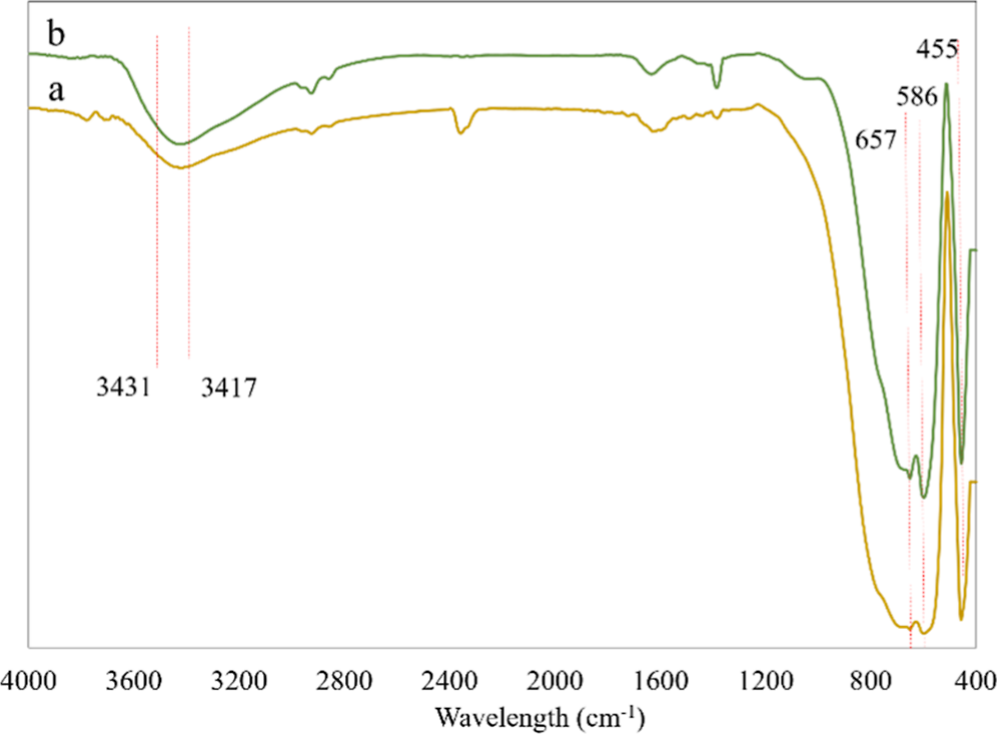
FTIR spectra at range 4000–400 cm^–1^ (a) before and (b) after Cr adsorption process using FeAl adsorbents.

Figure 1A,B illustrate the FTIR spectra of all composites within two spectral regions (A) 4000–3000 cm^–1^, and (B) 1000–400 cm^–1^, prior to Cr adsorption. The incorporation of Fe, Co, and Ni onto Al_2_O_3_ disrupted the bands in all adsorbents indicating structural modifications in the alumina framework. Key absorption bands observed include the broad –OH stretching at 3417 cm^–1^, Al–O–Al vibration at 455 cm^–1^ and metal (M–O–M) lattice vibrations within 657–586 cm^–1^, confirming the presence of metal-incorporated alumina frameworks consistent with literature.^33^ The broad absorption band at 3417 cm^–1^ was attributed to intermolecular and intramolecular hydrogen bonding of –OH groups, indicating surface hydroxylation, which plays a key role in metal ion adsorption.^34^ Upon Fe, Co, and Ni incorporation, the increased intensity of the –OH band suggests enhanced hydroxyl functionalization, with FeAl showing the most pronounced broadening –OH stretching band indicating enhanced hydroxylation and higher Al–OH surface density.^35^ The Al–O–Al peak at 455 cm^–1^ remained present but showed reduced sharpness, suggesting slight distortion of the alumina lattice due to metal substitution.^36^ Furthermore, the M–O–M stretching at 657–586 cm^–1^ intensified in metal-loaded composites, suggesting successful isomorphous substitution and Al–O-M bond formation.^37,38^ All of the peaks intensify, possibly due to the conversion of Al–O–Al to Al–O-M. Meanwhile, the isomorphous substitution of Fe, Co, and Ni ions with hydrogen atoms from Al_2_O_3_ hydroxyl nests resulted in Al–O-M species.

Figure 2a,b display FTIR spectra of FeAl before and after the adsorption of Cr ions, revealing significant spectral shifts indicative of adsorbent–adsorbate interactions. It was observed that FeAlCr exhibited bands that were associated with –OH stretching at 3431 cm^–1^, Al–O–Al at 455 cm^–1^ and M–O–M stretching in the range of 657–597 cm^–1^. The peaks revealed that the –OH stretching at 3417 cm^–1^ shifted to 3431 cm^–1^ after adsorption, indicating structural rearrangements and enhanced electrostatic interactions between Cr ions and surface hydroxyl groups.^39,40^ At 657–597 cm^–1^, the M–O–M stretching band decreased in intensity due to the isomorphous substitution of metals in the composites. FTIR analysis confirms structural modifications in FeAl, CoAl, and NiAl after metal incorporation. These changes enhance surface hydroxylation and alter the electronic environment of the alumina matrix. Shifts in –OH and M–O–M bands after Cr adsorption highlight hydrogen bonding, electrostatic interactions, and metal substitution in adsorption. These findings affirm FeAl’s superior adsorption efficiency and magnetic separability for heavy metal removal.

### Crystallinity

Figure 3 depicts the wide-angle XRD patterns of bare Al, NiAl, CoAl, and FeAl, synthesized using the PM method, within the 2θ range of 20°–80°. The diffraction peaks for Al appear at 2θ = 27.05°, 37°, 39.2°, 45°, 58.9°, and 67.1°, corresponding to the conventional cubic structure of Al_2_O_3_ (JCPDS 10–0425).^41–43^

**Figure 3:**
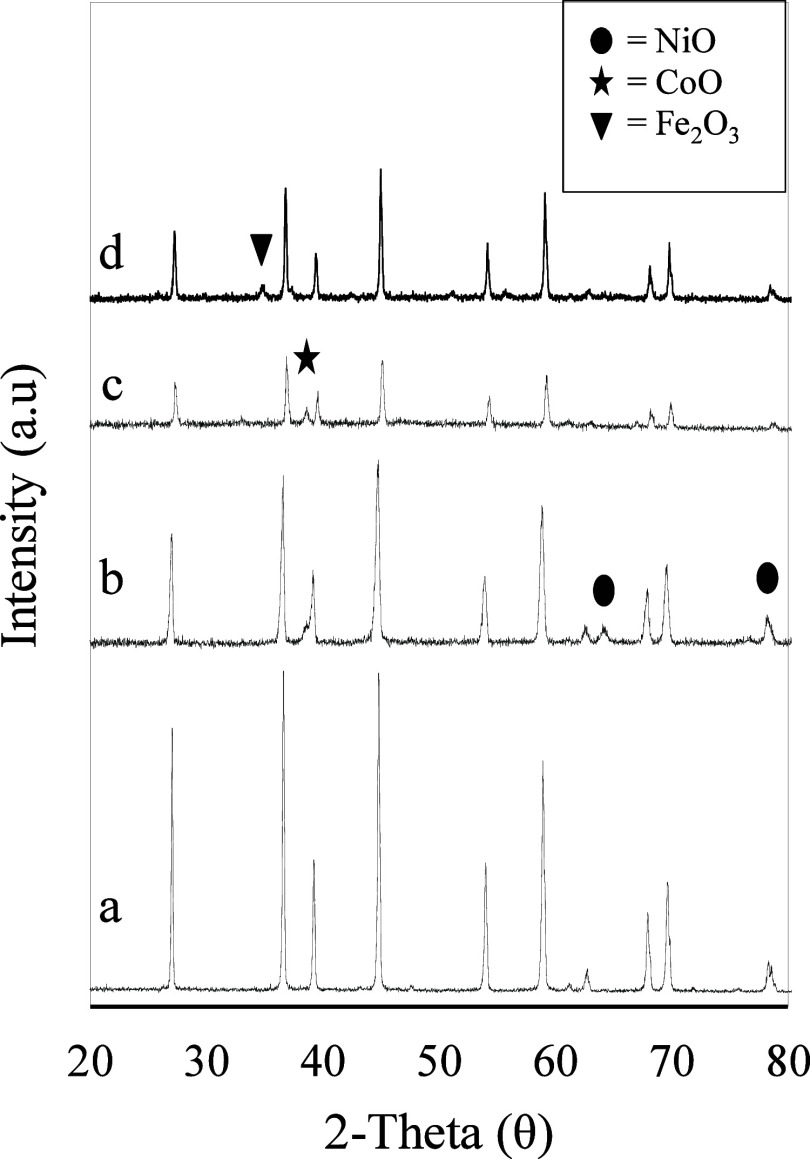
XRD pattern of adsorbents (a) Al, (b) NiAl, (c) CoAl, (d) FeAl.

The incorporation of Fe, Co, and Ni into the Al_2_O_3_ framework resulted in a reduction in Al peak intensity, suggesting structural modifications and possible metal incorporation into the alumina lattice. Prior research has attributed this peak reduction to changes in crystallinity upon metal integration.^44^ Distinct diffraction peaks at 63° (JCPDS 78–643) and a weak peak at 78° (JCPDS 47–1049) confirm the presence of NiO phases in the NiAl composite.^45^ Similarly, peaks near 39° indicate the formation of CoO in CoAl,^42^ while a peak at 34.9° confirms the hematite phase with rhombohedral symmetry (JCPDS 33–0664) in FeAl.^46^ The presence of these characteristic peaks in Figure 3b–d further verifies the successful incorporation of metal oxides into the Al_2_O_3_ support.

The absence of distinct metal phases in some regions of the XRD spectra suggests either high dispersion of metal oxides within the Al_2_O_3_ matrix or low metal loading concentrations, rendering them undetectable by XRD.^47^ The effective dispersion of metal oxides within the Al_2_O_3_ support is crucial for enhancing the adsorption efficiency in Cr ion removal. Similar peak broadening and metal oxide integration were reported for Fe-doped silica and biochar composites,^17,21^ but their diffraction peak intensities often remain lower due to weaker crystallinity or incomplete incorporation. In contrast, the defined hematite phase and reduced Al_2_O_3_ peaks in FeAl highlight a more effective structural transformation supporting enhanced adsorption. The XRD analysis conclusively confirms the formation of Al_2_O_3_, NiO, Fe_2_O_3_, and CoO phases, reinforcing the structural integrity and potential of FeAl-based adsorbents for wastewater treatment applications.

### Morphological and Textural Studies

SEM photographs of FeAl and quantitative results are presented in Figure 4. Figure 4 reveals FeAl’s structure as a highly porous structure with an irregular shape, which is comprised of Fe and Al. The observed morphology is consistent with previous studies in Fe-modified alumina composites.^43^ Specifically, Al has a cylindrical shape, while Fe particles displayed a quasi-spherical morphology, consistent with findings by Pan et al., who reported the formation of hollow α-Fe_2_O_3_ spheres.^48^ Figure 4 clearly confirmed the successful synthesis of nanosized FeAl composites with particle sizes ranging from 20 to 50 nm.

**Figure 4:**
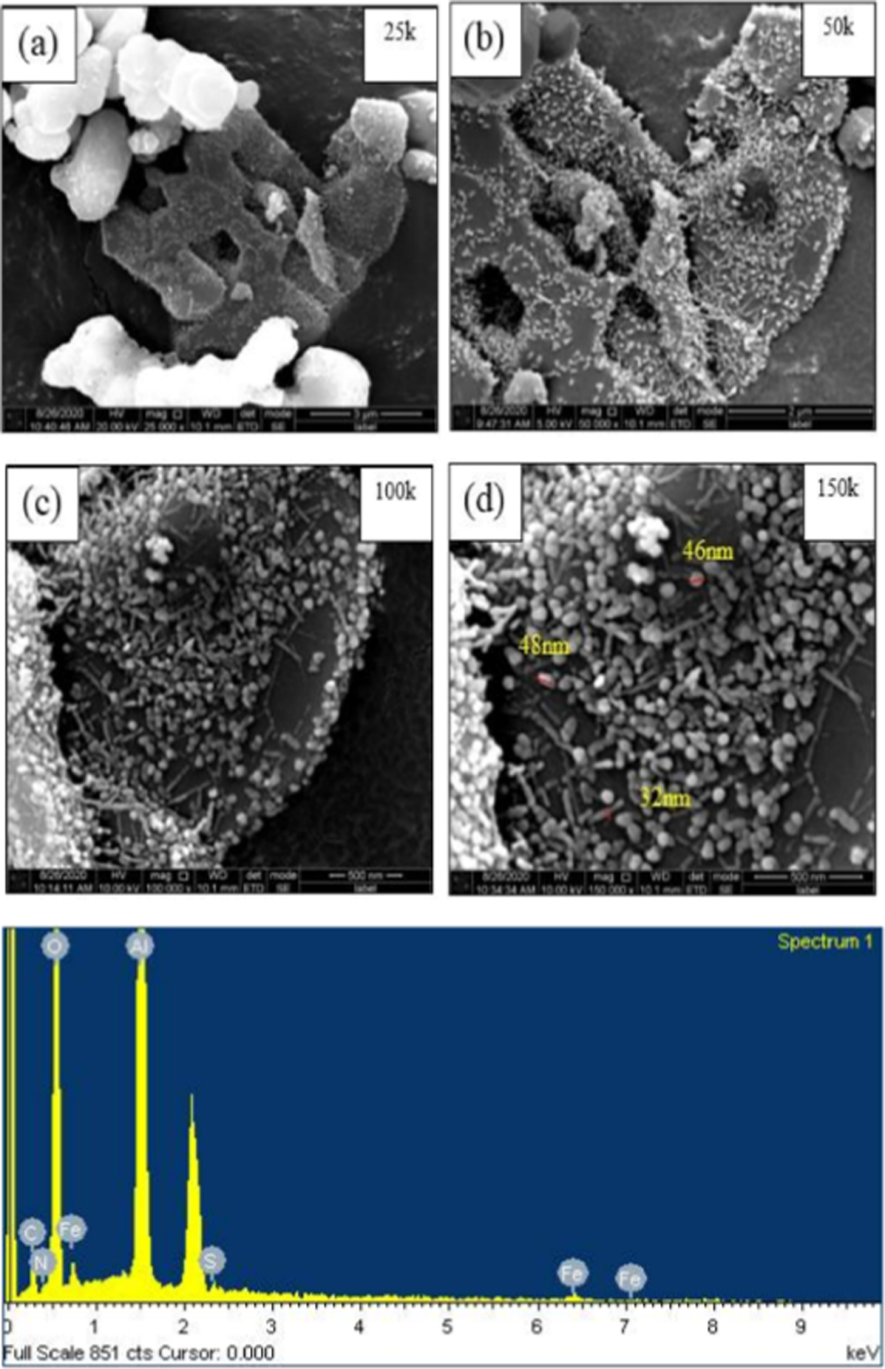
SEM images at (a) 2k, (b) 5k, (c) 100k, and (d) 150k magnification and EDX of FeAl.

Meanwhile, EDX spectral analysis, as visualized in Figure S1, verified the elemental composition, indicating a Fe/Al ratio of 10:90, confirming the effective incorporation of Fe within the alumina matrix. These findings provide strong evidence of homogeneous Fe dispersion, which is critical for optimizing adsorption performance and enhancing surface reactivity.

The nitrogen (N_2_) adsorption–desorption isotherms of Al, FeAl, CoAl, and NiAl as illustrated in Figure S2 confirm the mesoporous nature of all synthesized adsorbents, with Al and NiAl exhibiting nearly complete pore filling and emptying, indicative of well-defined porosity.^49^ According to the BET classification, all the samples exhibit a type IV isotherm, which is characteristic of condensation and evaporation steps for typical mesoporous materials.^50^ The relative pressure range (P/P_o_) = 0.3–0.4 means the internal mesopores, P/P_o_ = 0.4–0.7 represents the porosity within the uniform channels, and P/P_o_ = 0.8–1.0 represents the textural porosity resulting from the noncrystalline intra-aggregate voids and spaces formed by interparticle contact.^51^

The presence of H1 hysteresis loops in all adsorbents suggests the formation of highly uniform cylindrical mesopores, where capillary condensation occurs within P/P_0_ = 0.6–1.0.^34^ Notably, the desorption process of Al and NiAl nearly superimposes the adsorption process, leading to smaller adsorption loops compared to FeAl and CoAl, which display broader hysteresis loops, indicative of enhanced pore connectivity and structural stability.^52^ The textural properties, including surface area, pore volume, and pore diameter, are summarized in Table 1, providing quantitative insights into the structural differences among Al, NiAl, CoAl, and FeAl.

**Table 1:** Textural Properties of the Adsorbents

adsorbent	Al	NiAl	CoAl	FeAl
surface area (m^2^ g^–1^)	0.665	1.252	1.858	2.486
pore volume (mL g^–1^)	0.004	0.011	0.017	0.024
pore diameter (nm)	38.41	30.61	34.17	296.15

The observed variations in pore structure and adsorption behavior suggest that magnetic incorporation of the Ni, Co, and Fe significantly influences the porosity and surface characteristics of the alumina-based adsorbents compared to the bare Al, which in turn affects their adsorption efficiency and performance in heavy metal remediation. The pore size of FeAl is the largest, measuring 296.15 nm compared to other composites. This is attributed to isomorphous substitution-driven pore expansion, where the contraction of alumina walls leads to pore coalescence and improved textural porosity.^53^ The high surface area and expanded pore volume further enhance FeAl’s adsorption potential, facilitating efficient metal ion diffusion and stability during the adsorption process. In contrast, the smaller pore diameters of NiAl and CoAl, despite their higher surface area, suggest pore-blocking effects due to metal substitution, which restrict the available adsorption sites.^45,53^

Eventually, Figure S2 reveals a monomodal profile, with peak diameters ranging between 100 and 300 nm, confirming the mesoporous nature of all composites. In summary, the SEM study successfully demonstrated the irregular shape and nanosize range of 20–50 nm for FeAl, a size comparable to magnetic gamma alumina nanoparticles. Meanwhile, the BET analysis confirmed its type-IV mesoporosity, highlighting its suitability as an efficient adsorbent for Cr ion removal.^31^

### Oxygen Vacancies

XPS analysis was conducted to examine the phase structure and OV in the adsorbent material. Figure 5 depicts the XPS spectra of the adsorbents, highlighting the essential spinel compounds and confirming their existence in the samples. The XPS spectra of FeAl before (a) and after (b) Cr adsorption are shown in Figure 5, covering 60–130 eV, 705–735 eV, and 380–680 eV.

**Figure 5:**
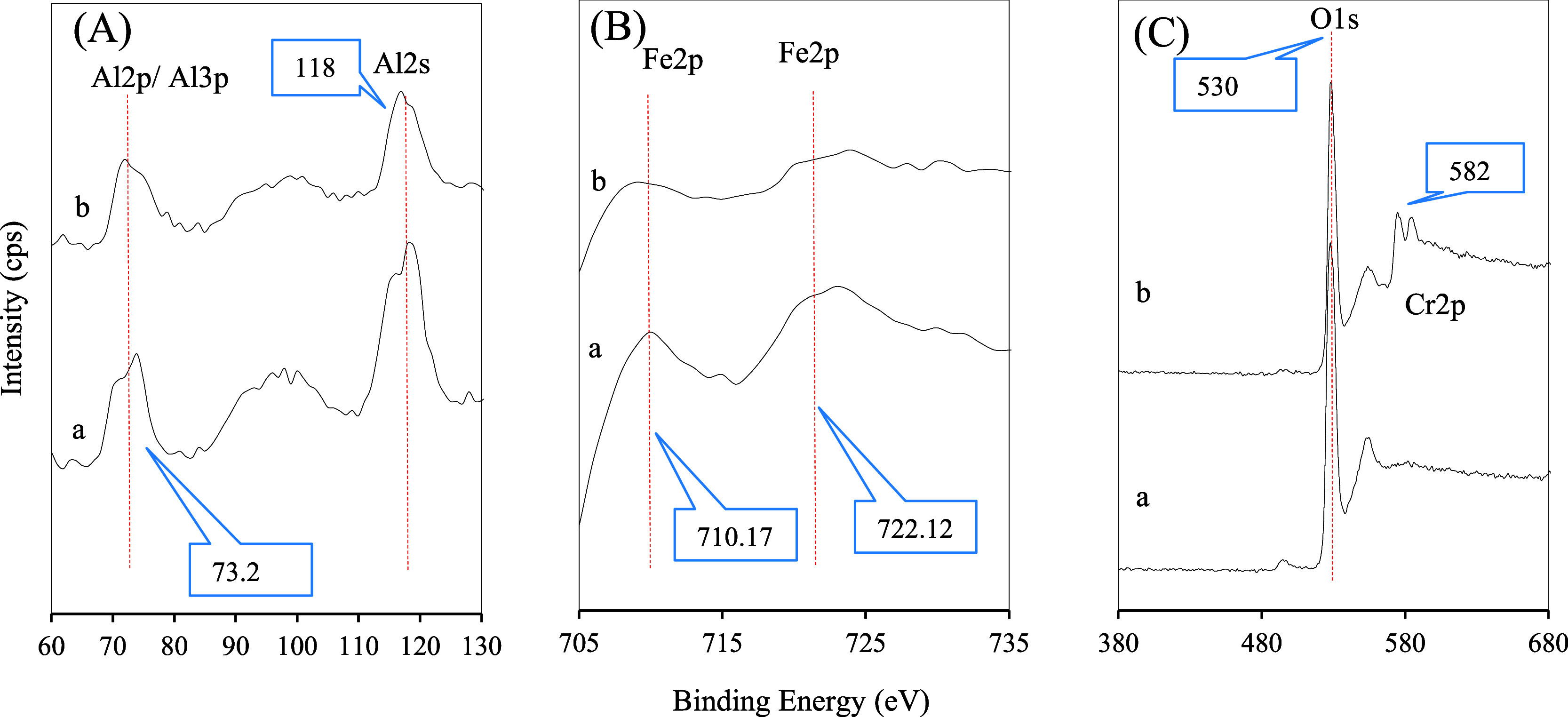
XPS spectra before adsorption (a) FeAl, and after adsorption (b) FeAlCr, in the region (A) 60–130 eV, (B) 705–735 eV and (C) 380–680 eV.

Broad peaks for Al 2p at 73.2 eV and Al 2s at 118 eV were visualized in Figure 5a, representing the Al bayerite structure, characteristic of the alumina structure.^54^ After Cr adsorption, Figure 5A­(b) reveals a decrease in Al_2_O_3_ peak intensity, suggesting Al dealumination and phase transformation due to calcination. In Figure 5B­(a), Fe 2p peaks are observed at 710.17 and 722.17 eV, which correspond to Fe 2p 3/2 and Fe 2p 1/2, consistent with previously reported values of 711.65 and 724.85 eV.^55^ The oxygen peaks are interpreted for O 1s at 530 eV, illustrated in Figure 5C, shifting to higher binding energy after Cr adsorption, indicative of dealumination, isomorphous substitution, and the replacement of hydrogen atoms on the adsorbent surface.

At the same time, Figure 5C also reveals that the peaks around 582 eV are related to Cr metal ions, and the decrease in the intensity of Al peaks demonstrates the occurrence of isomorphous substitution.^56^ Furthermore, the XPS spectra of Fe 2p and O 1s confirm that Fe and O exist in +3 and −2 valence states, respectively.^57^ Here, the findings suggest that Cr adsorption promotes the formation of spinel and oxide on the FeAl support, contributing to enhanced adsorption performance and surface reactivity.

### Magnetization

ESR analysis was conducted to analyze the electromagnetic radiation absorption by unpaired electrons The ESR spectra for FeAl, CoAl, NiAl, and Al are shown in Figure 6. These findings reinforce the role of OV in influencing the electronic environment and magnetic behavior of the adsorbents, further correlating with their adsorption performance.

**Figure 6:**
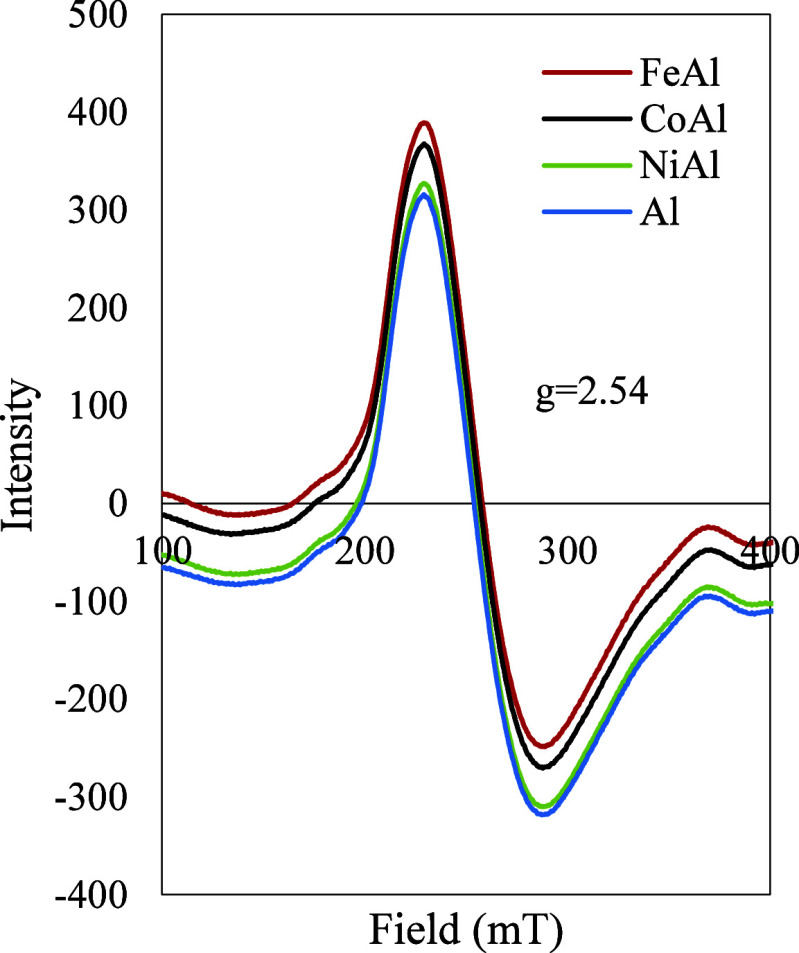
ESR spectra of FeAl, CoAl, NiAl, Al.

At g = 2.54 lines, the spectra of all adsorbents were recorded, explaining the random dispersion of Fe, Co, and Ni over the Al surface.^58^ Changes in ESR signal intensity explained the OV concentrations in the samples. The highest ESR signal intensity was observed in FeAl, indicating a significant presence of OVs, which is indicative of a higher frequency of unpaired electrons compared to other composites. These vacancies create additional active sites and enhance electron mobility, thereby strengthening surface interactions with Cr ions and ultimately increasing the adsorption efficiency compared to the other composites. In contrast, the smallest ESR signal was recorded for Al, confirming a weak paramagnetic setting with an unpaired electron.^59^ The reduced signal for CoAl and NiAl implied isomorphous metal substitution into the Al framework, which preserved the charge neutrality of that weak contact.^60^ Furthermore, NiAl exhibits an ESR signal nearly equivalent to Al, which can be attributed to the stabilizing effect of paired electrons within the Ni–Al framework. Meanwhile, CoAl displays a lower OV concentration, likely due to weaker paramagnetic interactions compared to FeAl. Additionally, the hydrogen spillover effect at the adsorbent sites may have contributed to OV saturation with electrons, further modulating the ESR response.^29^

### Zeta Potential

Zeta potential analysis as in Figure S3 revealed that magnetic metal incorporation significantly modulates surface charge, enhancing Cr^3+^ adsorption performance. Al exhibited a slightly positive surface charge of +7.5 mV, which decreased marginally to +3.2 mV after Cr^3+^ adsorption, suggesting weak electrostatic interaction. In contrast, FeAl, CoAl, and NiAl composites displayed markedly more negative zeta potentials of −26.3 mV, −18.7 mV, and −14.2 mV respectively indicating improved surface reactivity. Upon Cr^3+^ uptake, all composites became less harmful, with FeAl showing the most considerable shift of 10.9 mV, consistent with strong electrostatic attraction and possible inner-sphere complexation. By comparison, previous study has reported that Fe_3_O_4_ and chitosan-lignosulfonate composites typically retain more negative postadsorption zeta potentials of −22.5 mV at similar pH, indicating weaker surface charge compensation and less efficient electrostatic interaction with Cr^3+^.^61^ The strong surface charge, combined with magnetic separability, addresses key limitations in conventional nonmagnetic adsorbents.

### Adsorption of Cr Ion

The adsorption of Cr ions was studied using bare Al and its composite adsorbents: FeAl, CoAl, NiAl, FeAl­(C), CoAl­(C), and NiAl­(C). The effects of contact time, solution pH, adsorbent dosage, initial Cr concentration, and temperature were systematically evaluated to optimize adsorption performance and understand the adsorption mechanism. A batch adsorption study was initially conducted at different contact times (30, 60, 90, 120, 150, 180, and 210 min) by taking an initial concentration of 10 mg L^–1^ Cr ions with 0.02 g of each adsorbent at pH 5 and 30 °C. The adsorption percentage as a function of contact time is illustrated in Figure S3. According to Figure S4, FeAl exhibited the highest adsorption percentage, reaching 16% initially, with a significant increase over time. Equilibrium was attained at 150 min, with 35% Cr ion adsorption, and the percentage continued to rise beyond this point. The other adsorbents followed a similar trend, eventually reaching equilibrium but without exceeding the FeAl adsorption value. The findings revealed that adsorbents synthesized via the PM method demonstrated higher adsorption capacities compared to those synthesized via the CP method, with bare Al exhibiting the lowest adsorption rate. Moreover, PM method can be considered more feasible for scale-up, as it requires fewer reagents, lower energy input, and simpler processing steps compared to CP method and similar research.^23,28^ Based on these results, FeAl was identified as the most effective adsorbent and was selected for further parameter assessments. The pH value of the aqueous solution is a crucial parameter to consider during an adsorption study. The adsorption of Cr ions onto FeAl was evaluated across a pH range of 3 to 11 ^3,5,7,9,11^ for a 150 min contact time. Figure S5 illustrates the effect of pH on Cr adsorption using FeAl for 150 min, with an initial Cr concentration of 10 mg L^–1^, an adsorbent dosage of 0.02 g, and a temperature of 30 °C. The results confirmed that Cr adsorption is pH-dependent. At pH 3, the adsorption efficiency was reduced due to the high concentration of positively charged hydronium ions (H^+^) and Cr^3+^ ions, leading to electrostatic repulsion and strong competition for active sites.^62,63^ At higher pH levels, the formation of insoluble metal hydroxides contributed to additional interactions during the adsorption process.^64^ Compared to pH 5, adsorption was significantly higher at pH 7, indicating that neutral conditions favor Cr removal. FeAl’s optimal adsorption at pH 7 aligns with industrial wastewater conditions, thereby reducing the need for pH adjustments and improving cost efficiency for large-scale applications.

Adsorbent dosage is a key factor in optimizing adsorption efficiency. The adsorption of Cr ions was studied by varying FeAl dosages from 0.01 to 0.06 g, using a pH 7 aqueous solution, an initial Cr concentration of 10 mg L^–1^, and a contact time of 150 min at 30 °C. Figure S6 presents the adsorption percentage of Cr as a function of FeAl dosage. Figure S5 indicates that increasing the adsorbent dosage enhances the adsorption efficiency, attributed to the increased surface area and availability of active sites. Adsorption percentages peaked at 0.05 g, beyond which the adsorption efficiency declined at 0.06 g, likely due to a low ratio of adsorption surface sites to adsorbent. Since most Cr ions were already bound to the adsorbents, additional adsorbent mass did not significantly enhance removal efficiency. Accordingly, for a 10 mg L^–1^ Cr solution, 0.05 g of FeAl was determined to be the optimal dosage for further adsorption studies. Consequently, the optimum initial concentration for adsorption efficiency was determined by continuing Cr adsorption experiments with 0.05 g of FeAl.

The impact of initial Cr concentration (10–100 mg L^–1^) on adsorption was evaluated using 0.05 g of FeAl at pH 7 and 30 °C for 150 min. Figure S7 presents the adsorption percentage at different concentrations. At 10 mg L^–1^, Cr ion removal increased rapidly, reaching 75% at 25 mg L^–1^ as observed in Figure S6. As adsorption progressed, the adsorption percentage gradually decreased. At lower concentrations, the abundance of active adsorption sites enabled effective removal of Cr. However, at higher concentrations, the limited availability of active sites became insufficient, resulting in saturation and a decline in the adsorption percentage. This saturation occurs due to the high competition among Cr ions for binding sites, preventing complete removal at elevated concentrations. Based on these findings, 25 mg L^–1^ was determined as the optimal initial Cr concentration, and this parameter was used for subsequent experiments.

Temperature is a crucial factor in adsorption studies as it influences the diffusion rate, adsorbate–adsorbent interactions, and overall adsorption capacity. Adsorption experiments were performed at 30 °C, 40 °C, and 50 °C, maintaining constant conditions of pH 7, 150 min contact time, 0.05 g FeAl, and 25 mg L^–1^ Cr concentration. Figure S8 illustrates the effect of temperature on adsorption percentage which indicates that Cr adsorption increased from 30 to 40 °C, reaching a maximum efficiency of 91% at 40 °C. This enhancement is attributed to increased molecular motion and improved collision frequency, which facilitate Cr ion interactions with active sites.^63,65^ However, at 50 °C, the adsorption percentage declined, likely due to excessive thermal agitation weakening the adsorbate–adsorbent interactions, leading to Cr desorption. The adsorption percentage first increases when the temperature rises from 30 to 40 °C and then starts decreasing with a further increase in temperature from 40 to 50 °C.^66^ Considering the ideal temperature for the wastewater industry was between 30 and 40 °C, the findings demonstrated significant optimum conditions for Cr adsorption at 40 °C.

### Isotherm Modeling

Isotherm analysis was employed to forecast the adsorption capacity and surface interaction of FeAl, providing critical insights for optimizing the adsorption system. Figure S9 and Table 2 illustrate the linear plots of different isotherm models and the extracted data. The adsorption equilibrium was evaluated using Langmuir, Freundlich, Temkin, and Dubinin–Radushkevich isotherm models to assess the nature of Cr ion interactions with FeAl.

**Table 2:** Isotherm Study

Langmuir		Freundlich		Temkin		Dubinin–Radushkevich	
*q*_m_ (mg g^–1^)	500.0	*K*_F_ (mg g^–1^)/ (L mg^–1^)^1/n^	2.91	*K*_T_ (L mg^–1^)	0.634	*q*_m_	115
*K*_L_ (L mg^–1^)	0.0046	*n*_F_	1.13	*B*_T_	39.2	*K*_D_ (mol^2^ kJ^–2^)	0.00003
*R* ^2^	0.9899	*R* ^2^	0.975	*R* ^2^	0.873	E	183
*R*_L_	0.6839					*R* ^2^	0.79

### Langmuir Isotherm

The Langmuir model illustrates the adsorption mechanism of the sorbent’s monolayer coverage. Figure S9A displays linear correlation plots between the variables 1/*q*_e_ and Ce, showing the relationship between q_e_ and C_e_. The adsorption of Cr ions onto the selected adsorbent was well described by the Langmuir isotherm model, indicating monolayer adsorption on a homogeneous surface with a saturation of active sites at higher concentrations. The strong linear correlation (*R* ^2^ = 0.9899) between 1/q_e_ and C_e_ confirms the model’s suitability and reliability in predicting adsorption behaviors. The Langmuir constant, *K*_L_, derived from the slope and intercept of the linearized plot, represents both the adsorbent capacity and energy of adsorption. The *R* ^2^ values were used to determine the optimal linear equation. Langmuir explained Cr ion adsorption onto FeAl best, with the highest *R* ^2^ accuracy among the other three models. The correlation coefficient (*R* ^2^ = 0.9899) indicates a strong fit of the Langmuir isotherm model to the experimental data for FeAl. The adsorption affirms that adsorption occurs at specific, uniform sites without lateral interactions, with a maximum *q*_m_ of 500.0 mg g^–1^ compared to the reported study using MnO_2_ colloidal particles with a *q*_m_ of 144.5 mg g^–1^.^67^ Another parameter, known as the “separation factor” or “equilibrium parameter”, *R*_L_, was utilized to assess the favorability of adsorption in the Langmuir isotherm model. *R*_L_ values are classified as unfavorable (*R*_L_ > 1), linear (*R*_L_ = 1), favorable (0 < *R*_L_ < 1), or irreversible (*R*_L_ = 0). The *R*_L_ value of 0.6839 falls within the favorable range, indicating that the adsorption process is both feasible and spontaneous.

### Freundlich Isotherm

The Freundlich isotherm model was employed to characterize the adsorption behavior of Cr ions on the adsorbent, particularly emphasizing surface heterogeneity and multilayer adsorption. The model is represented by the linearized equation, where ln *q*_e_ is plotted against ln Ce as visualized in Figure S9B, allowing the determination of the Freundlich constants n_F_ and *K*_F_ from the slope and intercept, respectively. The relatively high *R* ^2^ value (*R* ^2^ = 0.975) suggests a good correlation, reinforcing the model’s suitability for describing the adsorption mechanism. A crucial parameter in the Freundlich isotherm, the heterogeneity factor (*n*_F_), provides insight into the nature of adsorption, whether the adsorption is linear (*n*_F_ = 1), chemical (*n*_F_ < 1), or physical (*n*_F_ > 1). The *n*_F_ value derived from this study was 1.13, as presented in Table 2, suggesting that the adsorption process predominantly follows a physical adsorption mechanism, which is deemed favorable as per the established range of 1 < *n*_F_ < 10.^68^

### Temkin Isotherm

The Temkin isotherm model was depicted based on two assumptions: (i) the heat of adsorption decreases linearly due to the presence of adsorbent–adsorbate interactions for all molecules in the layer, and (ii) adsorption is identified by a uniform distribution of binding energies, up to a specific maximum energy.^65^ The bT parameter, which was positive and small, indicates weak interactions between Cr ions and the adsorbent, confirming a physical adsorption and exothermic nature of the process.^69^ A moderate linear correlation (*R* ^2^ = 0.873) from the Temkin linear plot, as shown in Figure S9C, suggests that while the model captures variations in adsorption energy, it does not fully describe the mechanism compared to other models, such as the Langmuir and Freundlich models.

### Dubinin–Radushkevich Isotherm

The DR isotherm model was applied to evaluate the porosity and adsorption energy distribution of the adsorbent. Unlike Langmuir and Freundlich models, which primarily describe adsorption equilibrium, the DR model provides insights into the adsorption mechanism by calculating the mean sorption energy (*E*). The magnitude of E distinguishes between physisorption (*E* < 8 kJ mol^–1^) and chemisorption (*E* > 16 kJ mol^–1^), offering a quantitative assessment of adsorption energy.^71^ eq 7 was used to calculate the E value.

7 $$E=\frac{1}{\sqrt{2K_{DR}}}$$

Contrary to the Temkin and Freundlich results, the *E* value of 182.6 kJ mol^–1^ estimated for FeAl suggests adsorption follows a chemisorption mechanism, characterized by strong ionic or covalent bonding between Cr ions and the adsorbent. The high *E* value suggests that adsorption is not solely a surface phenomenon, but rather involves penetration into the adsorbent’s microporous structure and potential surface complexation mechanisms. Since the DR model assumes adsorption onto a heterogeneous surface, the results indicate that adsorption occurs in a multistep process, combining physical interactions at initial binding sites with strong chemical bonding as adsorption progresses. The linear correlation (*R* ^2^ = 0.79) from the DR model indicates a moderate fit to the experimental data. While the model effectively captures the adsorption energy distribution effectively, the lower *R* ^2^ value suggests that adsorption is not entirely governed by pore-filling mechanisms alone and may involve additional surface interactions. The maximum monolayer capacity (*q*_m_) of 500 mg g^–1^ for FeAl surpasses values reported for most magnetic or biosorbents, which typically range from 80 to 300 mg g^–1^.^7^ This is primarily due to the synergistic effect of magnetic substitution and mesoporous structure, enabling enhanced Cr affinity and selectivity.

Kinetic study. The adsorption kinetics were investigated to determine the rate and mechanism of Cr ion uptake. The Lagergren pseudo-first-order and Ho pseudo-second-order models were applied using eqs 8 and ([Disp-formula eq9]), respectively. The data were plotted as illustrated in Figure S10 and then analyzed using the Weber and Morris Intraparticle diffusion model to determine the possible mechanism and rate-determining step of adsorption based on eq 10.

8 $$Pseudo-first\;order,q_{t}=q_{e}\left(1-e^{-k1t}\right)$$

9 $$Pseudo-second\;order,q_{t}=\frac{q_{e}^{2}k_{2}t}{1+q_{e}k_{2}t}$$

10 $$Intra\;particle\;diffusion,q_{t}=k_{id}t^{1/2}+C_{i}$$

Where q_e_ is the amount of Cr ions adsorbed per gram of adsorbent (mg g^–1^); q_t_ is the adsorption capacity acquired at time t (mg g^–1^); t is the time interval (min); and *k*_1_ and *k*_2_ are pseudo-first (min^–1^) and pseudo-second order rate constants (mg g^–1^ min^–1^), respectively. *k*_id_ is a constant of intraparticle diffusion (mg g^–1^ min^–1/2^), and C_i_ is the thickness of the boundary layer.

The pseudo-first-order model assumes that adsorption is proportional to available sites, while the pseudo-second-order model suggests chemisorption as the dominant process. Kinetic parameters and R^2^ are summarized in Table 3, demonstrating that both kinetic models fit the experimental data.

**Table 3:** Kinetic Study of Cr

		adsorbent dosage (g)				
		0.02	0.03	0.04	0.05	0.06
*q*_e,exp_ (mg g^–1^)	33.8	29	24.4	21.9	17	
pseudo-first-order	*k*_1_ × 10^–2^ (min^–1^)	2.44	2.56	2.67	2.49	2.58
	*q*_e, cal_ (mg g^–1^)	28.7	26	21.8	19	16.2
	*R* ^2^	0.981	0.963	0.971	0.978	0.996
pseudo-second-order	*k*_2_ x10^–3^ (g mg^–1^ min^–1^)	13.3	15	19.7	20.1	20.8
	*q*_e, cal_ (mg g^–1^)	37.5	32.3	26.9	24.3	19.3
	*R* ^2^	0.999	0.998	0.999	0.998	0.998
intraparticle diffusion	K_id_ x 10^–1^ (mg g^–1^ min^–1^)	22.6	19.4	16.2	14.6	11.7
	C_i_ (mg g^–1^)	5.87	5.07	4.43	3.82	2.47
	*R* ^2^	0.879	0.88	0.87	0.88	0.905

To further visualize the adsorption process, the relationship between q_t_ and time is presented in Figure S10. As shown in Table 3, the *R* ^2^ indicate a good fit for both the pseudo-first-order and pseudo-second-order kinetic models. However, the pseudo-first-order model provided q_e_ values closer to the experimental data, particularly at higher adsorbent dosages. For instance, at 0.06 g FeAl, the calculated *q*_e_ of 16.2 mg g^–1^ is closer to the experimental *q*_e_ of 17.0 mg g^–1^, while the pseudo-second-order model is higher than experimental at 19.3 mg g^–1^. This evidence suggests that the adsorption process primarily follows physisorption, which is consistent with the pseudo-first-order kinetics. Pseudo-first order q_e_ values were more in line with the experimental results than pseudo-second order q_e_ values. These findings suggest that the physisorption mechanism is favored by the first-order kinetic models for FeAlCr.^69^

To further investigate the adsorption mechanism, the Weber and Morris intraparticle diffusion model was used to evaluate mass transfer limitations. Figure S10 showed that intraparticle diffusion was the rate-limiting step, as indicated by the first line, representing ion mass transfer, which grew significantly at first before gradually slowing down in the last figure. Moreover, Table 3 revealed a downward trend in *q*_e_, *K*_id_, and *C*_i_ with increasing adsorbent dosage. Due to the increased availability of the adsorption surface at higher dosages, the boundary layer became thinner. Subsequently, the intraparticle diffusion rate slowed due to the increasing molecule-to-molecule collisions. As a result of the boundary layer being thinner under these conditions, the physisorption process of adsorption was facilitated.

### Thermodynamic Study

Thermodynamic analysis was conducted at 30 °C, 40 °C, and 50 °C to evaluate the nature and feasibility of the adsorption process. Generally, elevating the temperature of the adsorption process enhances the adsorption capacity due to increased contaminant mobility in the aqueous solution, which increases the accessibility of the adsorption sites. The thermodynamic parameters; Gibbs free energy (Δ*G*), enthalpy (Δ*H*), entropy (Δ*S*), and activation energy (*E*a) were determined using eq 11 – ([Disp-formula eq14]), with values presented in Table 4. The linear form of the Van’t Hoff from eq 11 was plotted as shown in Figure S11 to calculate the values of Δ*H* and Δ*S* from the slope and intercept of ln *K*_c_ versus 1/T, respectively.

11 $$\ln K_{c}=\frac{\Delta S^{{}^{\circ}}}{R}-\frac{\Delta H^{{}^{\circ}}}{RT}$$

12 $$K_{c}=\frac{C_{e}\left(adsorbent\right)}{C_{e}\left(solution\right)}$$

13 $$\Delta G=-RT\ln K_{c}$$

14 $$E_{a}=k_{\mathrm{int}}t^{1/2}+C$$

**Table 4:** Thermodynamic Study

temp (K)	*q*_e,exp_ (mg g^–1^)	Δ*G* (kJ mol^–1^)	Δ*H* (kJ mol^–1^)	Δ*S* (kJ mol-1 K^–1^)	*E*_a_ (kJ mol-1 K^–1^)
303	56.3	–2.77	–26.6	–0.0994	95.9
313	68.3	–6.02			
323	63.8	–4.66			

Adsorption can be either exothermic or endothermic depending on the sign of Δ*H*. Positive values of Δ*H* imply the adsorption process is endothermic, whereas negative values indicate an exothermic process.^70,71^ As stated in Table 4, the negative Δ*H* value of −26.6 kJ mol^–1^ for FeAlCr suggests that the adsorption process is exothermic, consistent with the bT parameter from the Temkin model. Additionally, the absolute values of Δ*H* indicate whether the process is physisorption (<24 kJ mol^–1^) or chemisorption (>24 kJ mol^–1^).^72^ Considering Δ*H* for FeAlCr was greater than 24 kJ mol^–1^, the adsorption proceeds via chemisorption, involving strong enthalpic contributions from electrostatic interactions, rather than weak van der Waals forces.

The negative Δ*S* of −0.0994 J mol^–1^ K^–1^ for FeAlCr implies a reduction in system disorder upon adsorption. The decrease is likely due to the restricted mobility of Cr ions upon adsorption, a characteristic of surface complexation and a strong binding affinity, further supporting a chemisorption mechanism. Meanwhile, the Δ*G* determines the spontaneity of the adsorption.^73,74^ For FeAlCr, the negative values of Δ*G* indicate spontaneous, exothermic adsorption, with higher temperatures reducing adsorbate–adsorbent affinity.

The *E*_a_ is another critical factor in determining the adsorption mechanism. eq 14 employs the Arrhenius equation and the pseudo-first-order kinetics rate constant *k*_1_ to calculate *E*_a_. Since the *E*_a_ for FeAlCr was 95.9 kJ mol^–1^ which is greater than 40 kJ mol^–1^, the adsorption mechanism is confirmed to be chemisorption, requiring a significant energy input to initiate the process. The positive sign indicated that more energy is required to initiate the adsorption process, and vice versa. The ion exchange for mesoporous materials was explained by a range of 0.2–80 kJ mol^–1^.^71,72^

### Reusability

The reusability of FeAl was examined over five sequential adsorption–desorption cycles. As illustrated in Figure S12, the Cr ion removal efficiency slightly declined from 91.0% in the first cycle to 83.8% in the fifth. The minimal loss in performance highlights FeAl’s structural robustness, attributed to its stable Fe–O–Cr coordination, strong electrostatic interaction, and retention of surface hydroxyl groups, as evidenced by similar degradation patterns reported in the literature, where active-site fouling and functional group loss were identified as key contributors to reusability loss in metal-modified adsorbents.^74,75^ These results suggest FeAl is a reliable and reusable adsorbent, suitable for multiple cycles in practical wastewater treatment applications.

Mechanism of Cr adsorption. Adsorbent structures and processes of metal ion adsorption onto FeAl were presented. Figure 7 depicts the mechanism of Cr adsorption by FeAl, highlighting the isomorphous substitution of the metal and dealumination of Al.

**Figure 7:**
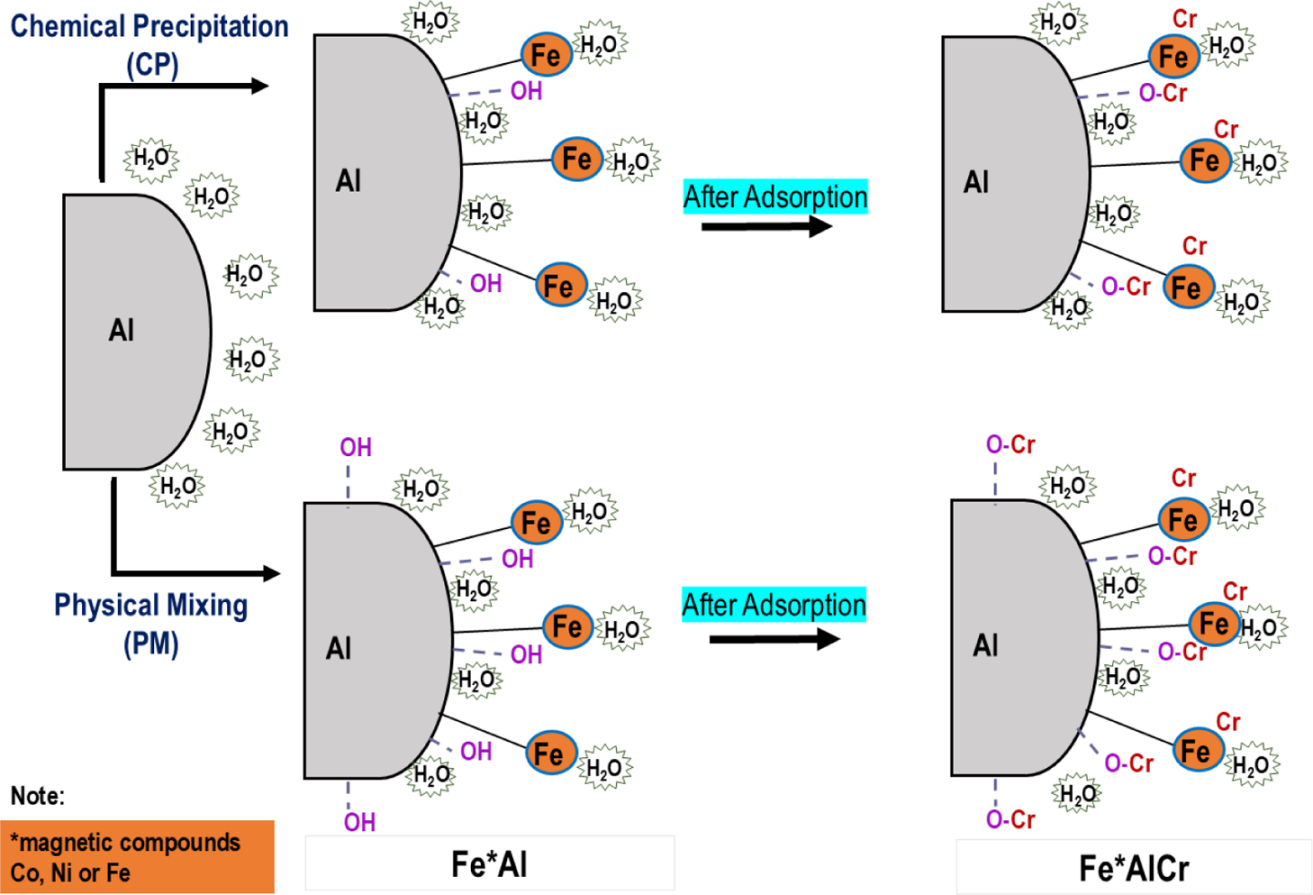
Proposed mechanism for adsorption of FeAl and FeAl (C).

The roles of oxygen vacancies and isomorphous substitution have been further clarified to strengthen the structure–property interpretation. Oxygen vacancies increase local electron density and create defect-rich active sites capable of binding Cr species, while isomorphous substitution of Fe, Co, and Ni into the alumina lattice perturbs the electronic environment and enhances surface charge modulation. These combined effects intensify electrostatic attraction and facilitate inner-sphere complexation, explaining the markedly superior performance of FeAl.

Bare Al displayed low adsorption due to its limited surface area, minimal reactive sites, and scarcity of functional groups. Metal incorporation via PM or CP introduced substantial physicochemical modifications that enhanced Cr uptake, as validated by FTIR, SEM-EDX, BET, XRD, XPS, and ESR analyses. FTIR analysis as in Figures 1 and 2, showed that FeAl exhibited the broadest and most intense—OH stretching band at 3417 cm^–1^, corresponding to high hydroxyl density. These – OH groups enable electrostatic attraction and ligand exchange with Cr ions, explaining the higher reactivity of FeAl compared to FeAl­(C). Enhanced M–O–Al bands at 678–586 cm^–1^ further indicate lattice modification following metal incorporation. The decrease in Al peak intensity of XRD results, as illustrated in Figure 3, suggesting structural distortion that generates new adsorption-active sites. XPS as observed in Figure 5, confirmed the presence of Fe_2_O_3_, NiO, and CoO phases and showed decreased Al signal intensity after adsorption, evidencing Fe–O–Cr coordination and surface complexation. Meanwhile, BET analysis revealed that FeAl possessed the highest surface area of 2486 m^2^ g^–1^, pore volume of 0.024 mL g^–1^, and mesopore diameter with 296.15 nm, improving ion diffusion and increasing the accessibility of active sites. FeAl’s higher porosity relative to FeAl­(C) correlates directly with its improved adsorption capacity. Additionally, ESR measurements at *g* = 2.54 revealed that metal ions were randomly dispersed across the Al surface, increasing OV density and magnetism, key factors that enhanced Cr adsorption. These vacancies generate additional adsorption-active centers and enhance electron transfer, supporting FeAl’s superior performance compared to CoAl and NiAl.

While previous studies^13,76,77^ have proposed electrostatic interaction or ligand exchange as dominant mechanisms, FTIR, XPS, and ESR data support a multipathway adsorption mechanism involving Fe–O–Cr complexation, confirmed by XPS, hydroxyl enhancement with support by FTIR, OV-driven electron enrichment, indicated by ESR; and electrostatic attraction, dominant near neutral pH where FeAl exhibits a strongly negative zeta potential when compared to other composites The strongest ESR signal in FeAl suggests a higher density of reactive sites, further supporting its superior adsorption efficiency compared to CoAl and NiAl, which exhibited weaker ESR signals.

### Conclusion

This study successfully developed a novel FeAl composite via a simple and scalable physical mixing route, demonstrating markedly enhanced Cr adsorption performance. Fe incorporation significantly increased surface hydroxylation, oxygen-vacancy density, and enlarged mesopore diameter (296.15 nm), and strong Fe–O–Cr coordination, all of which strengthened the surface–adsorbate interactions. These structural enhancements resulted in a high adsorption capacity of 500 mg g^–1^ and 91% removal efficiency under optimized conditions of pH 7, 40 °C and 25 mg L^–1^ Cr ion concentration. Adsorption followed the Langmuir model (R^2^ = 0.9899) and pseudo-first-order kinetics, confirming monolayer uptake dominated by electrostatic attraction and Fe–O–Cr surface complexation. Thermodynamic analysis indicated a spontaneous and exothermic adsorption with partial chemisorption (Δ*H* = – 26.6 kJ mol, *E*_a_ = 95.9 kJ mol^–1^). FeAl retained 83.8% efficiency after five cycles, indicating good structural stability and reusability potential. Overall, these findings support its potential for practical application in industrial wastewater treatment and provide a foundation for future work on multimetal systems and dynamic flow applications. Future research should focus on further on quantifying magnetic separation efficiency, evaluating recovery performance across multiple cycles, correlating magnetization strength with practical recyclability and scaling up synthesis for industrial applications.

## Supplementary Material



## Notes

EDX spectrum, N_2_ adsorption–desorption isotherms and pore size distribution curve; batch adsorption study, fitting parameters of isotherm models, adsorption kinetic and thermodynamic study and reusability (PDF)

## Abbreviations

AAS,: atomic absorption spectroscopy
Al,: alumina
BET,: brunauer–Emmett–Teller
CoAl,: cobalt alumina
CP,: chemical precipitation
EDX,: energy-dispersive X-ray
ESR,: electron spin resonance
FeAl,: iron alumina
FTIR,: fourier transform infrared spectroscopy
NiAl,: nickel alumina
PM,: physical mixing
SEM,: scanning electron microscope
XPS,: X-ray photoelectron spectroscopy
XRD,: X-ray diffraction.

## References

[1] Yang H., Huang K., Zhang K., Weng Q., Zhang H., Wang F. (2021). Predicting
Heavy Metal Adsorption on Soil with Machine Learning and
Mapping Global Distribution of Soil Adsorption Capacities. Environ. Sci. Technol..

[2] Yang A. M., Lo K., Zheng T. Z., Yang J. L., Bai Y. N., Feng Y. Q., Cheng N., Liu S. M. (2020). Environmental Heavy Metals and Cardiovascular
Diseases: Status and Future Direction. Chronic
Dis. Transl. Med..

[3] Mitra S., Chakraborty A. J., Tareq A. M., Emran T. B., Nainu F., Khusro A., Idris A. M., Khandaker M. U., Osman H., Alhumaydhi F. A., Simal-Gandara J. (2022). Impact of
Heavy Metals on The Environment and Human Health: Novel Therapeutic
Insights to Counter the Toxicity. Journal of
King Saud University—Science.

[4] Obasi P. N., Akudinobi B. B. (2020). Potential Health Risk and Levels
of Heavy Metals in
Water Resources of Lead–Zinc Mining Communities of Abakaliki,
Southeast Nigeria. Appl. Water Sci..

[5] Wang X.-L., Guo D.-M., An Q.-D., Xiao Z.-Y., Zhai S.-R. (2019). High-Efficacy
Adsorption of Cr­(VI) And Anionic Dyes Onto Β-Cyclodextrin/Chitosan/Hexamethylenetetramine
Aerogel Beads with Task-Specific, Integrated Components. Int. J. Biol. Macromol..

[6] Yang H.-R., Yang C., Li S.-S., Shan X.-C., Song G.-L., An Q.-D., Zhai S.-R., Xiao Z.-Y. (2022). Site-Imprinted Hollow
Composites with Integrated Functions for Ultra-Efficient Capture of
Hexavalent Chromium from Water. Sep. Purif.
Technol..

[7] Prasad S., Yadav K. K., Kumar S., Gupta N., Cabral-Pinto M. M. S., Rezania S., Radwan N., Alam J. (2021). Chromium Contamination
and Effect on Environmental Health and Its Remediation: A Sustainable
Approaches. J. Environ. Manage..

[8] C F. C., Kamalesh T., Kumar P. S., Rangasamy G. (2023). A Critical
Review on the Sustainable Approaches for the Removal of Toxic Heavy
Metals from Water Systems. Ind. Eng. Chem. Res..

[9] Ahmed S. F., Mehejabin F., Momtahin A., Tasannum N., Faria N. T., Mofijur M., Hoang A. T., Vo D.-V. N., Mahlia T. M. I. (2022). Strategies
to Improve Membrane Performance in Wastewater Treatment. Chemosphere.

[10] Sepahdar A., Almasian A., Javar H. A. (2021). Functionalized
Carbon/Alumina/Silica
Nano-Fibrous Membrane: Preparation, Characterization and Heavy Metal
Filtration Performance. Desalin. Water Treat..

[11] Hussain M., Ali V., Pourebrahimi S., Ahmadi S., Ghosh S. (2023). Chemical Methods of
Heavy Metal Management: Coagulation, Flocculation, And Floatation. ACS Symp. Ser..

[12] El-Sawaf A. K., El-Dakkony S. R., Zayed M. A., Eldesoky A. M., Nassar A. A., El Shahawy A., Mubarak M. F. (2024). Green Synthesis and Characterization
of Magnetic Gamma Alumina Nanoparticlesfor Copper Ions Adsorption
from Synthetic Wastewater. Results Eng..

[13] Kumar A., Das T., Thakur R. S., Fatima Z., Prasad S., Ansari N. G., Patel D. K. (2022). Synthesis of Biomass-Derived
Activated Carbons and
Their Immobilization on Alginate Gels for the Simultaneous Removal
of Cr­(VI), Cd­(II), Pb­(II), As­(III), and Hg­(II) from Water. ACS Omega.

[14] Li H., Zheng F., Wang J., Zhou J., Huang X., Chen L., Hu P., Gao J.-M., Zhen Q., Bashir S., Liu J. L. (2020). Facile Preparation of Zeolite-Activated
Carbon Composite from Coal Gangue with Enhanced Adsorption Performance. Chem. Eng. J..

[15] Farghali M. A., Abo-Aly M. M., Salaheldin T. A. (2021). Modified
Mesoporous Zeolite-A/Reduced
Graphene Oxide Nanocomposite for Dual Removal of Methylene Blue and
Pb^2+^ Ions from Wastewater. Inorg.
Chem. Commun..

[16] Joshi P., Raturi A., Srivastava M., Khatri O. P. (2022). Graphene Oxide,
Kaolinite Clay And PVA-Derived Nanocomposite Aerogel as A Regenerative
Adsorbent for Wastewater Treatment Applications. J. Environ. Chem. Eng..

[17] Aziz M. A. A., Jalil A. A., Triwahyono S., Mukti R. R., Taufiq-Yap Y. H., Sazegar M. (2014). Sazegar. Highly Active Ni-Promoted Mesostructured Silica
Nanoparticles for CO_2_ Methanation. Appl. Catal. B Environ. Energy.

[18] Yaseen M., Ullah S., Ahmad W., Subhan S., Subhan F. (2021). Fabrication
of Zn and Mn Loaded Activated Carbon Derived from Corn Cobs for The
Adsorptive Desulfurization Of Model and Real Fuel Oils. Fuel.

[19] Khir N. N. H. M., Muhamad Salleh N. F., Ghafar N. N. A., Shukri N. N. M. (2024). Mitigating
Health Risks Through Biosorption: Effective Removal of Nickel (II)
And Chromium (VI) From Water with Acid-Treated Potato Peels. Jurnal Kesehatan Lingkungan..

[20] salah
omer A., A El Naeem G., Abd-Elhamid A. I., O M Farahat O., A El-Bardan A., M A Soliman H., Nayl A. A. (2022). Adsorption of Crystal
Violet and Methylene Blue Dyes Using a Cellulose-Based Adsorbent from
Sugercane Bagasse: Characterization, Kinetic and Isotherm Studies. J. Mater. Res. Technol..

[21] Xiao F., Su D., Ren Y., Zhou J., Xu H., Li Z., He J. (2025). Efficient Removal of Cadmium (Cd^2+^) from Aqueous Solutions
by Chitosan@fig Branch Biochar: Adsorption Performance and Enhanced
Complexation. Langmuir.

[22] Li A. Y., Deng H., Jiang Y. H., Ye C. H., Yu B. G., Zhou X. L., Ma A. Y. (2020). MA. Superefficient
Removal of Heavy
Metals from Wastewater by Mg-Loaded Biochars: Adsorption Characteristics
and Removal Mechanisms. Langmuir.

[23] Zhu D., Chen Y., Yang H., Wang S., Wang X., Zhang S., Chen H. (2020). Synthesis
and Characterization of
Magnesium Oxide Nanoparticle-Containing Biochar Composites for Efficient
Phosphorus Removal from Aqueous Solution. Chemosphere.

[24] Xu M., Xu D., Zheng P., Liu H., Wang Y., Zhi Y. (2024). Phosphorus
Recovery and Heavy Metal Removal Potential from Sewage Sludge-Derived
Hydrochar with Activated Alumina. ACS Sustainable
Resour. Manage..

[25] Trang
Truong T. T., Tran T. D., Chi Tran T. K., Le T. A., Trang Luu T. H., Vu T. D., Nga N. K., Pham T. D. (2025). Adsorption
and Pre-Concentration of Fluoroquinolone Antibiotics In Surface Water
Using Novel Hybridized Ceo_2_/Al_2_O_3_ Nanocomposites. Microchem. J..

[26] Doan T. H. Y., Hoang T. H., Le V. A., Vu D. N., Vu N., Srivastav A. L., Pham T. D. (2022). Adsorption and Transformation of
Tetracyclines On Alpha Alumina Particles with Surface Modification
by Anionic Surfactant. Environ. Res..

[27] Huang Y., Peng Y., Zhang G., Wu Z., Li J., Ding W., Li H., An Y., Ao L., Shen Y., Zheng H. (2024). Synthesis and Fabrication of Magnetically
Separable Phosphate-Modified Magnetic Chitosan Composites for Lead­(II)
Selective Removal from Wastewater. Environ.
Res..

[28] Zhang M., Zhang S., Sun L., Li X., Chen H., Xu Q., Wang Z. (2020). One-Step Synthesis
Of 2-Mercaptobenzothiazole Functionalized
Magnetic Fe_3_O_4_ and Its Application for The Removal
of Heavy Metals. J. Taiwan Inst. Chem. Eng..

[29] Sun N., Wu Q., Jin L., Zhu Z., Sun J., Dong S., Xie H., Zhang C., Cui Y. (2023). Hyperbranched Magnetic Polymer: Highly
Efficient Removal of Cr­(VI) And Application In Electroplating Wastewater. Front. Chem. Sci. Eng..

[30] Ghorbani-Choghamarani A., Mohammadi M., Shiri L., Taherinia Z. (2019). Synthesis
and Characterization of Spinel Feal_2_o_4_ (Hercynite)
Magnetic Nanoparticles and Their Application in Multicomponent Reactions. Res. Chem. Intermed..

[31] Lu L., Li J., Ng D. H. L., Yang P., Song P., Zuo M. (2017). Synthesis
of Novel Hierarchically Porous Fe_3_O_4_@Mgal–LDH
Magnetic Microspheres and Its Superb Adsorption Properties of Dye
from Water. J. Ind. Eng. Chem..

[32] Vaithianathan R., Anitha P., Sudha R., Ramachandran A. (2023). Equilibrium
and Kinetic Studies on The Removal of Cadmium­(II) By Fe_3_O_4_ Loaded Activated Carbon Prepared from Castor Seed Shell. Desalin. Water Treat..

[33] İlktaç R., Bayir E. (2023). Magnetic Hydrogel
Beads as a Reusable Adsorbent for Highly Efficient
and Rapid Removal of Aluminum: Characterization, Response Surface
Methodology Optimization, And Evaluation of Isotherms, Kinetics, And
Thermodynamic Studies. ACS Omega.

[34] Zulfiqar N., Shariatipour M., Inam F. (2023). Sequestration of Chromium­(VI) And
Nickel­(II) Heavy Metals from Unhygienic Water Via Sustainable and
Innovative Magnetic Nanotechnology. Nanoscale
Adv..

[35] Wu C.-Y., Tu K.-J., Deng J.-P., Lo Y.-S., Wu C.-H. (2017). Markedly
Enhanced Surface Hydroxyl Groups of TiO_2_ Nanoparticles
with Superior Water-Dispersibility for Photocatalysis. Materials.

[36] Zhang Y., Zhang J. (2024). Synthesis of Ni, Cu
Plated Nano-Al_2_O_3_ Composite
Powders And Autocatalytic Mechanism. Mater.
Today Commun..

[37] Junaid M., Khan M. A., Hashmi Z. M., Nasar G., Kattan N. A., Laref A. (2020). Structural, Spectral,
Magnetic and Dielectric Properties of Bi Substituted
Li-Co Spinel Ferrites. J. Mol. Struct..

[38] Pang T., Yang X., Yuan C., Elzatahry A. A., Alghamdi A., He X., Cheng X., Deng Y. (2021). Recent Advance
in Synthesis and Application of Heteroatom Zeolites. Chin. Chem. Lett..

[39] Yaashikaa P. R., Palanivelu J., Hemavathy R. V. (2024). Sustainable
Approaches for Removing
Toxic Heavy Metal from Contaminated Water: A Comprehensive Review
of Bioremediation and Biosorption Techniques. Chemosphere.

[40] Yuan F., Yan D., Song S., Zhang J., Yang Y., Chen Z., Lu J., Wang S., Sun Y. (2025). Removal of Heavy Metals from Water
by Adsorption on Metal Organic Frameworks: Research Progress and Mechanistic
Analysis in The Last Decade. Chemical Engineering
Journal.

[41] Cirujano F. G., Martin N., Wee L. H. (2020). Design
of Hierarchical Architectures
in Metal–Oganic Frameworks for Catalysis and Adsorption. Chem. Mater..

[42] Singh A. K., Chen P.-W., Wuu D.-S. (2021). Growth and Characterization
of Co-Sputtered
Al-Doped Znga_2_o_4_ Films for Enhancing Deep-Ultraviolet
Photoresponse. Appl. Surf. Sci..

[43] Kusuma K. B., Manju M., Ravikumar C. R., Nagaswarupa H. P., Amulya M. A. S., Anilkumar M. R., Avinash B., Gurushantha K., Ravikantha N. (2020). Photocatalytic
and Electrochemical Sensor for Direct
Detection Of Paracetamol Comprising Γ-Aluminium Oxide Nanoparticles
Synthesized Via Sonochemical Route. Sens. Int..

[44] Gonçalves A. a. S., Costa M. J. F., Zhang L., Ciesielczyk F., Jaroniec M. (2018). One-Pot Synthesis Of MEAL_2_O_4_(ME
= NI, Co, Or CU) Supported On Γ-AL_2_O_3_with
Ultralarge Mesopores: Enhancing Interfacial Defects In Γ-AL2O3TO
Facilitate the Formation of Spinel Structures at Lower Temperatures. Chem. Mater..

[45] Wang T., Ma H., Zeng L., Li D., Tian H., Xiao S., Gong J. (2016). Highly Loaded Ni-Based
Catalysts For Low Temperature Ethanol Steam
Reforming. Nanoscale.

[46] Mao Z., Chen J., Yang Y., Wang D., Bie L., Fahlman B. D. (2017). Novel g-C_3_N_4_/CoO Nanocomposites
with Significantly Enhanced Visible-Light Photocatalytic Activity
for H_2_ Evolution. ACS Appl. Mater.
Interfaces.

[47] Pan W., Zhang Y., Yu S., Liu X., Zhang D. (2021). Hydrogen Sulfide
Gas Sensing Properties of Metal Organic Framework-Derived Α-Fe_2_O_3_ Hollow Nanospheres Decorated with Mose2 Nanoflowers. Sens. Actuators, B.

[48] Kordouli E., Pawelec B., Bourikas K., Kordulis C., Fierro J. L. G., Lycourghiotis A. (2018). Mo Promoted
Ni-Al2O3 Co-Precipitated
Catalysts for Green Diesel Production. Applied
Catalysis B Environment and Energy.

[49] Awan A., Niaz M., Saeed I., Raza Ma., Ahmad N., Atiq S., Riaz S., Naseem S. (2022). Magneto-Dielectric
Behavior of Electrodeposited FeAl_2_O_4_ Nanostructures. J. Mater. Sci.:Mater. Electron..

[50] Lellala K., Behera S. K., Srivastava P., Saeed W. S., Haidyrah A. S., Burile A. N. (2024). Fe_3_O_4_ Nanoparticles Decorated
On N-Doped Graphene Oxide Nanosheets for Elimination of Heavy Metals
from Industrial Wastewater and Desulfurization. Diamond Relat. Mater..

[51] Yagmur E., Gokce Y., Tekin S., Semerci N. I., Aktas Z. (2020). Characteristics
and Comparison of Activated Carbons Prepared from Oleaster (Elaeagnus
Angustifolia L.) Fruit Using KOH and ZnCl_2_. Fuel.

[52] Rechotnek F., Follmann H. D. M., Silva R. (2021). Mesoporous
Silica Decorated With
L-Cysteine as Active Hybrid Materials for Electrochemical Sensing
of Heavy Metals. J. Environ. Chem. Eng..

[53] Tao M., Zhou C., Shi Y., Meng X., Gu J., Gao W., Xin Z. (2020). Enhanced Sintering
Resistance of Bimetal/SBA-15 Catalysts
with Promising Activity Under a Low Temperature for CO Methanation. RSC Adv..

[54] Chong C. C., Abdullah N., Bukhari S. N., Ainirazali N., Teh L. P., Setiabudi H. D. (2019). Hydrogen Production Via CO_2_ Reforming of CH_4_ Over Low-Cost Ni/SBA-15 From Silica-Rich
Palm Oil Fuel Ash (POFA) Waste. Int. J. Hydrogen
Energy.

[55] Dinesh B. V. S., Bhattacharya A. (2020). Comparison of Energy Absorption Characteristics Of
PCM-Metal Foam Systems with Different Pore Size Distributions. J. Energy Storage.

[56] Jiang W., Zhang C., Feng Y., Wei B., Chen L., Zhang R., Ivey D. G., Wang P., Wei W. (2020). Achieving
High Structure and Voltage Stability in Cobalt-Free Li-Rich Layered
Oxide Cathodes Via Selective Dual-Cation Doping. Energy Storage Mater..

[57] Salleh N. F. M., Jalil A. A., Triwahyono S., Efendi J., Mukti R. R., Hameed B. H. (2015). New Insight into
Electrochemical-Induced Synthesis
of NiAl_2_O_4_/Al_2_O_3_: Synergistic
Effect Of Surface Hydroxyl Groups and Magnetism for Enhanced Adsorptivity
of Pd­(II). Appl. Surf. Sci..

[58] Zhu S., Zhang P., Liang Y., Wang M., Xiong J., Tan W. (2020). Effects Of Aluminum Substitution on The Surface Charge of Colloidal
Goethite Particles: Experiments and MUSIC Modeling. Environ. Sci. Pollut. Res..

[59] Jaafar N. F., Abdul Jalil A., Triwahyono S., Muhd Muhid M. N., Sapawe N., Satar M. A. H., Asaari H. (2012). Photodecolorization
of Methyl Orange Over Α-Fe_2_O_3_-Supported
HY Catalysts: The Effects of Catalyst Preparation and Dealumination. Chemical Engineering Journal.

[60] Chen C., Zhang H., Li K., Tang Q., Bian X., Gu J., Cao Q., Zhong L., Russell C. K., Fan M., Jia J. (2020). Cu^+^ Based
Active Sites of Different Oxides Supported Pd-Cu
Catalysts and Electrolytic In-Situ H_2_ Evolution for High-Efficiency
Nitrate Reduction Reaction. J. Catal..

[61] Davarcı D., Doğan N., Cabacı I. ˙., Zorlu Y. (2022). Manganese­(II), Cobalt­(II)
And Nickel­(II) Complexes Constructed from A Pyridyloxy-Functionalized
Hexapodal Cyclophosphazene Ligand: Structural and Magnetic Studies. Polyhedron.

[62] Alyasi H., Wahib S., Tong Y., Gomez T., Mahmoud K. A. (2025). Magnetic
MXene Chitosan-Lignosulfonate Composite (Fe_3_O4@ MCLS) for
the Reductive Removal of Cr­(VI) and Other Heavy Metals from Water. J. Hazard. Mater. Adv..

[63] Aziz F. F. A., Jalil A. A., Triwahyono S., Mohamed M. (2018). Controllable Structure
of Fibrous Sio2–ZSM-5 Support Decorated with TiO_2_ Catalysts for Enhanced Photodegradation of Paracetamol. Appl. Surf. Sci..

[64] Cisneros-Ontiveros H.
G., Medellín-Castillo N. A., Flores-Rojas A. I., Cruz-Briano S. A. (2024). Removal of Fluoride, Cadmium and
Triclosan from Water
Using Bone Chars from An Invasive Species: Optimization, Equilibrium
and Adsorption Mechanisms. Sustainable Chemistry
for the Environment.

[65] Sagar
Jena P., Pradhan A., Prakash Nanda S., Kishore Dash A., Naik B. (2022). Biosorption Of Heavy Metals from Wastewater Using Saccharomyces Cerevisiae
as a Biosorbent: A Mini Review. Mater. Today:
Proc..

[66] Ahamad Z., Mashkoor F., Nasar A., Jeong C. (2025). Multi-Walled Carbon
Nanotubes/Tio2/Chitosan Nanocomposite for Efficient Removal of Malachite
Green Dye from Aqueous System: A Comprehensive Experimental and Theoretical
Investigation. Int. J. Biol. Macromol..

[67] Subba
Reddy Y., Rotte N. K., Sudhakar B. K., Ramakrishna
Chand N., Naik R. J., Mandal S., Ravi Chandra M. (2024). Biomass-Derived
Sustainable Mesoporous Activated Carbon as An Efficient and Recyclable
Adsorbent for The Adsorption of Hazardous Dyes. Hybrid Advances.

[68] Tran T. M. H., Tran T. D., Dinh T. D., Nguyen M. K., Anh N. T. N., Nga N. K., Doan T. H. Y., Pham T. D. (2024). Adsorption Characteristics
Of Individual And Binary Mixtures Of Ciprofloxacin and Cr­(VI) In Water
Using Mno_2_ Colloidal Particles. Colloid
Polym. Sci..

[69] Selvaraj R., Murugesan G., Rangasamy G., Bhole R., Dave N., Pai S., Balakrishna K., Vinayagam R., Varadavenkatesan T. As (2022). Removal Using Superparamagnetic Magnetite Nanoparticles
Synthesised Using Ulva Prolifera – Optimization, Isotherm,
Kinetic and Equilibrium Studies. Chemosphere.

[70] Moghimi F., Jafari A. H., Yoozbashizadeh H., Askari M. (2020). Adsorption Behavior
of Sb­(III) In Single And Binary Sb­(III)­2014Fe­(II) Systems on Cationic
Ion Exchange Resin: Adsorption Equilibrium, Kinetic and Thermodynamic
Aspects. Trans. Nonferrous Met. Soc. China.

[71] Ahmad N., Suryani Arsyad F., Royani I., Lesbani A. (2022). Adsorption Of Methylene
Blue On Magnetite Humic Acid: Kinetic, Isotherm, Thermodynamic, And
Regeneration Studies. Results Chem..

[72] Ofudje E. A., Adeogun I. A., Idowu M. A., Kareem S. O., Ndukwe N. A. (2020). Simultaneous
Removals of Cadmium­(II) Ions and Reactive Yellow 4 Dye from Aqueous
Solution by Bone Meal-Derived Apatite: Kinetics, Equilibrium and Thermodynamic
Evaluations. J. Anal. Sci. Technol..

[73] Basit A., Yaqoob Z., Zahid A., Ali S., Shoukat B., Khaliq A., Chughtai M. T., Batul R., Rehman M. A. U., Husain S. W. (2025). Effective Adsorbent for The Removal
of Methylene Blue
Using Natural Serpentine /Magnetite Nanocomposites: Isotherm and Kinetic
Study. Heliyon.

[74] Yahuza S., Sabo I. A., Abubakar A., Shukor M. Y. (2021). Thermodynamic
Study
on The Biosorptive Removal of Lead (II) Ions from Aqueous Solutions
Using Acid-Treated Cystoseira Tricta Biomass. Journal of Environmental Microbiology and Toxicology.

[75] Juturu R., Vinayagam R., Murugesan G., Selvaraj R. (2025). Enhanced Adsorptive
Removal of Chromium (VI) Ions from Wastewater with Phosphorus-Doped
Magnetite-Carbon Composite: Advanced Statistical Physics Modeling
and Kinetic Studies. Environ. Dev. Sustain..

[76] Dharmapriya T. N., Li D., Chung Y.-C., Huang P.-J. (2021). Green Synthesis of Reusable Adsorbents
for The Removal of Heavy Metal Ions. ACS Omega.

[77] Hosseini S. G., Pasikhani J. V. (2019). Kinetic
and Thermodynamic Investigation on The Adsorption
of Hexavalent Chromium Pollution by Fe_3_O_4_/AC/Tio_2_ Nanotubes as A Novel Ternary Magnetic Nanocomposite. Desalin. Water Treat..

