# Supplementary material for: Magnetic Alumina for Chromium Ion Remediation: Isotherm Analysis and Kinetic Modeling
Source: ACS Omega. 2026 Apr 22;11(17):25946–59. doi: 10.1021/acsomega.6c01792 (PMC13150593; doi:10.1021/acsomega.6c01792)
Supplement: Supplementary file 1 [file ao6c01792_si_001.pdf]

Supporting Information

Magnetic Alumina for Chromium Ion Remediation: Isotherm Analysis and Kinetic Modeling

Siti Farimah Md Hanafiah[1], Northaqifah Hasna Mohamed Khir[1,2], Nur Fatien Muhamad Salleh[1], Nurasmat Mohd Shukri [1]**

[1]School of Health Sciences, Universiti Sains Malaysia, Health Campus, 16150 Kubang Kerian, Kelantan, Malaysia

[2]Faculty of Applied Sciences, Universiti Teknologi MARA, Terengganu Branch, Bukit Besi Campus, 23000 Dungun, Terengganu, Malaysia

Corresponding Author

*Nur Fatien Muhamad Salleh _ School of Health Sciences, Universiti Sains Malaysia, Health Campus, 16150 Kubang Kerian, Kelantan, Malaysi.; Email: nurfatiensalleh@usm.my

*Nurasmat Mohd Shukri_ School of Health Sciences, Universiti Sains Malaysia, Health Campus, 16150 Kubang Kerian, Kelantan, Malaysi.; Email: nurasmatms@usm.my

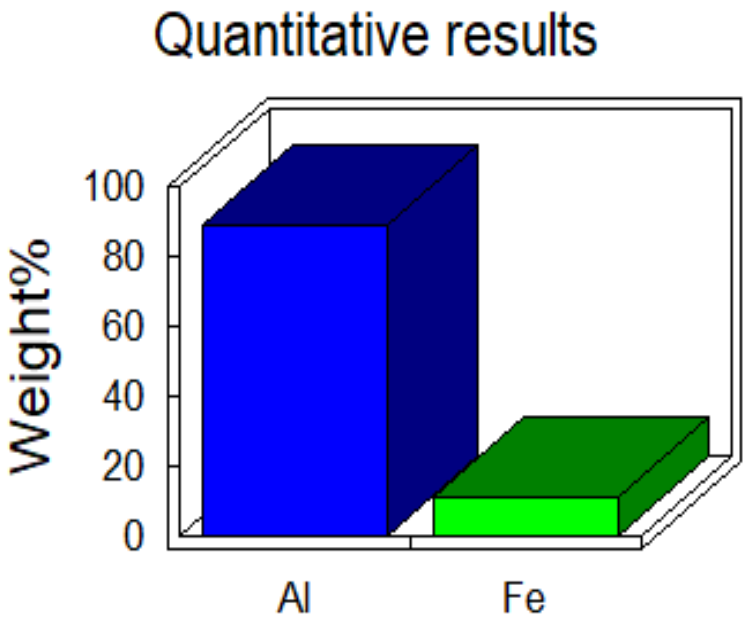

Figure S1. EDX Spectrum of FeAl

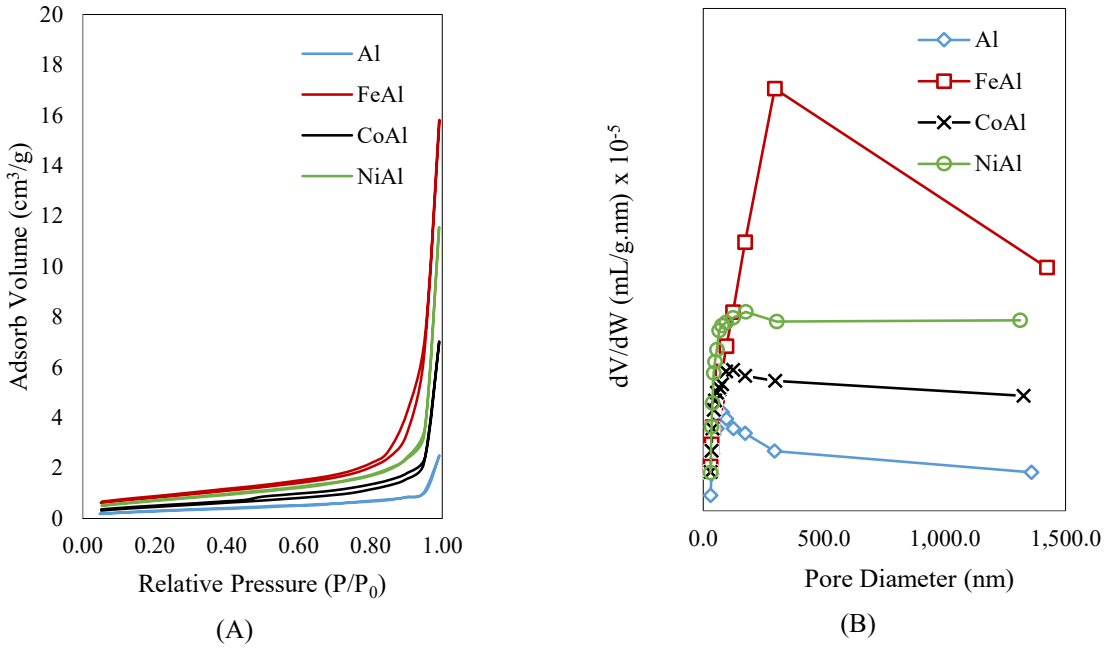

Figure S2. N_2 adsorption-desorption isotherms (a) BET isotherms and (b) pore size distribution of of Al, FeAl, CoAl, NiAl

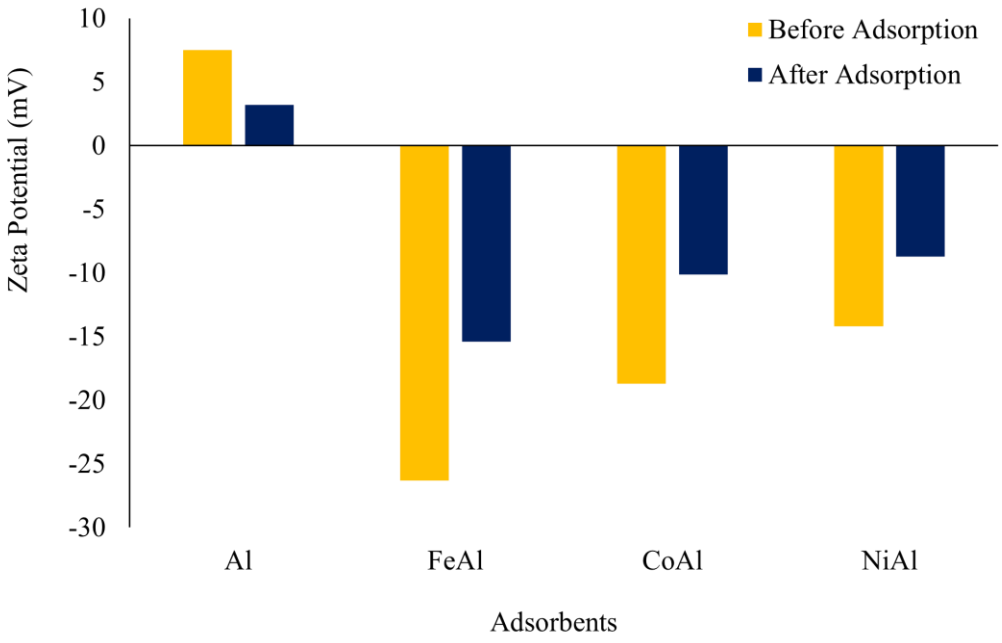

Figure S3. The zeta potential of all PM composites; Al, FeAl (PM), CoAl (PM) and NiAl (PM) before and after Cr ion adsorption.

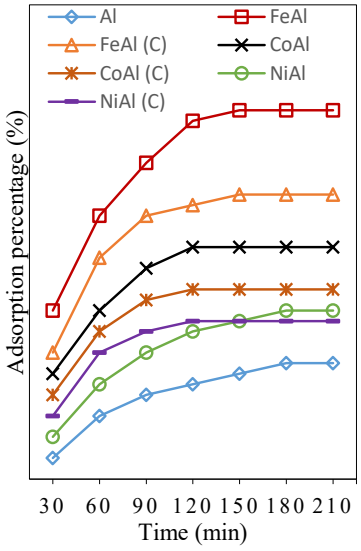

Figure S4. Adsorption percentage of 10 mg L^{-1} Cr ions as a function of contact time for all adsorbents; Al, FeAl, CoAl, NiAl, FeAl (C), CoAl (C), and NiAl (C) with 0.02 g of each adsorbent at pH 5 and 30°C

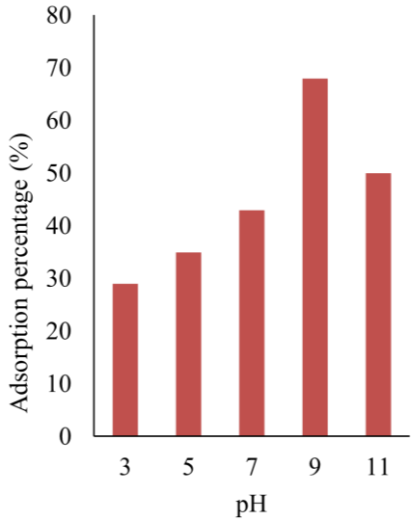

Figure S5. Effect of initial pH (3-11) on the adsorption percentage of 10 mg L^{-1} Cr ions using 0.02 g FeAl at temperature of 30 °C, and contact time of 150 minutes

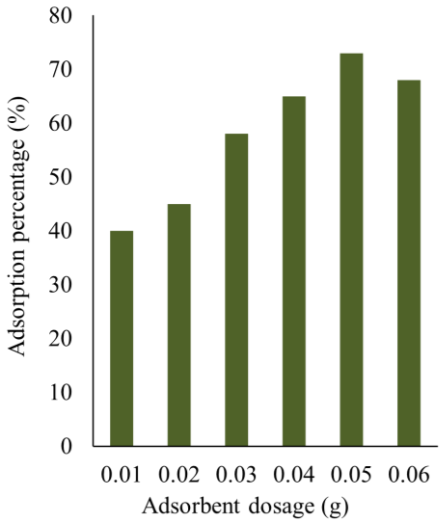

Figure S6. Effect of FeAl dosage (0.01-0.06 g) on the adsorption percentage of 10 mg L^{-1} Cr ions at pH 5, temperature of 30°C and contact time of 150 minutes.

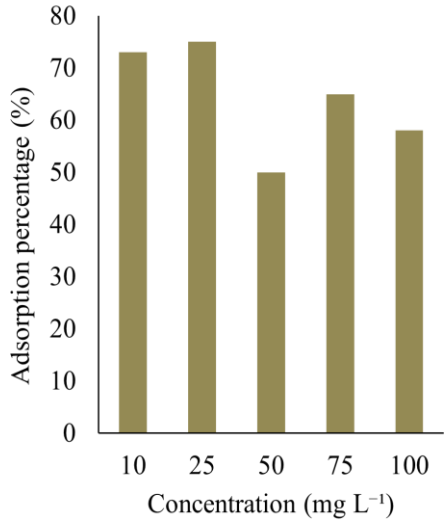

Figure S7. Effect of initial Cr ions concentration (10-100 mg L^{-1}) using 0.05 g FeAl on the adsorption percentage at pH 5, temperature of 30°C and contact time of 150 minutes

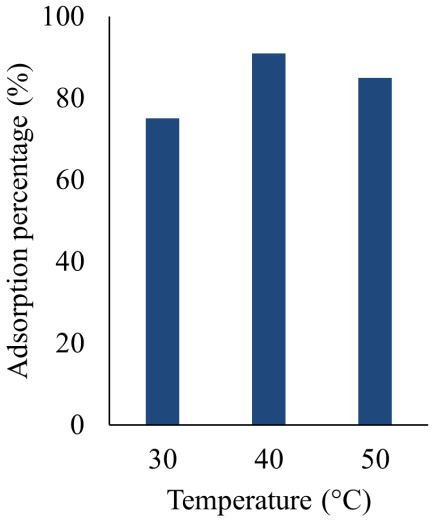

Figure S8. Effect of temperature (30-50 °C) using 0.05 g FeAl on the adsorption percentage of 25 mg L^{-1} Cr ions at pH 5, temperature of 30°C and contact time of 150 minutes.

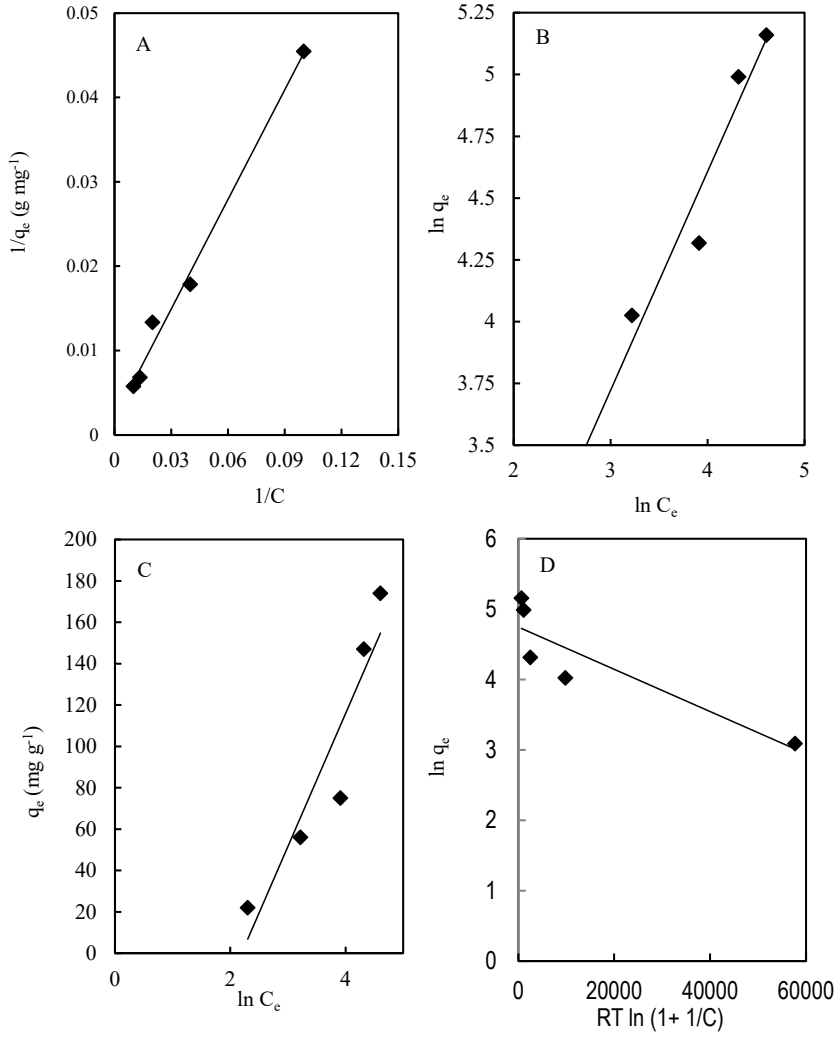

Figure S9. Isotherm study of FeAlCr, FeAlCd, FeAlMn: (A) Langmuir, (B) Freundlich, (C) Temkin, (D) Dubinin-Radushkevich

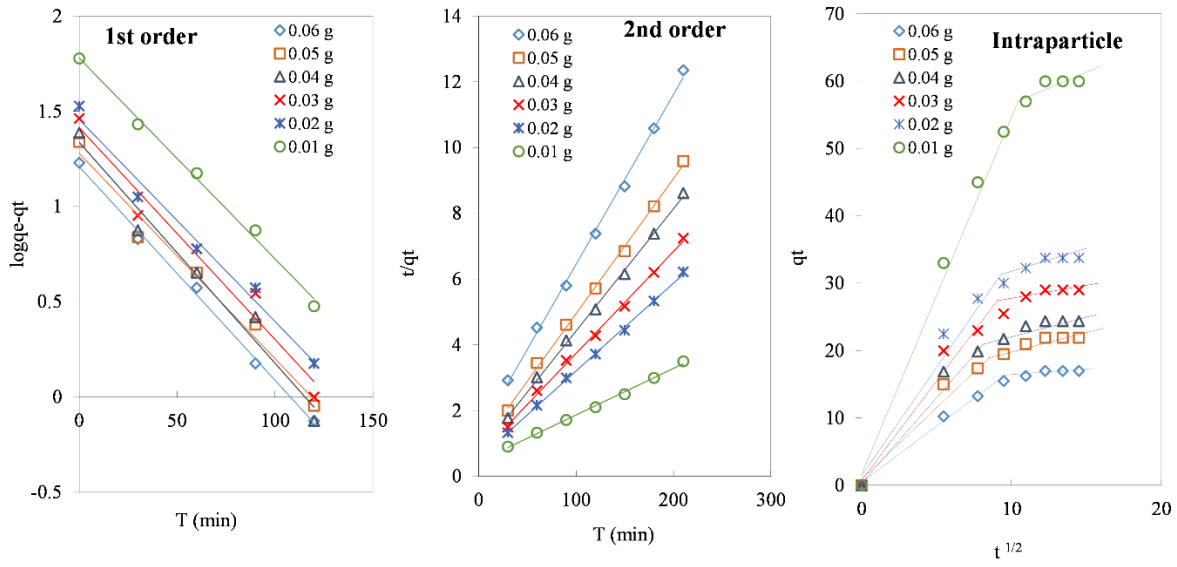

Figure S10. Kinetic study of FeAlCr

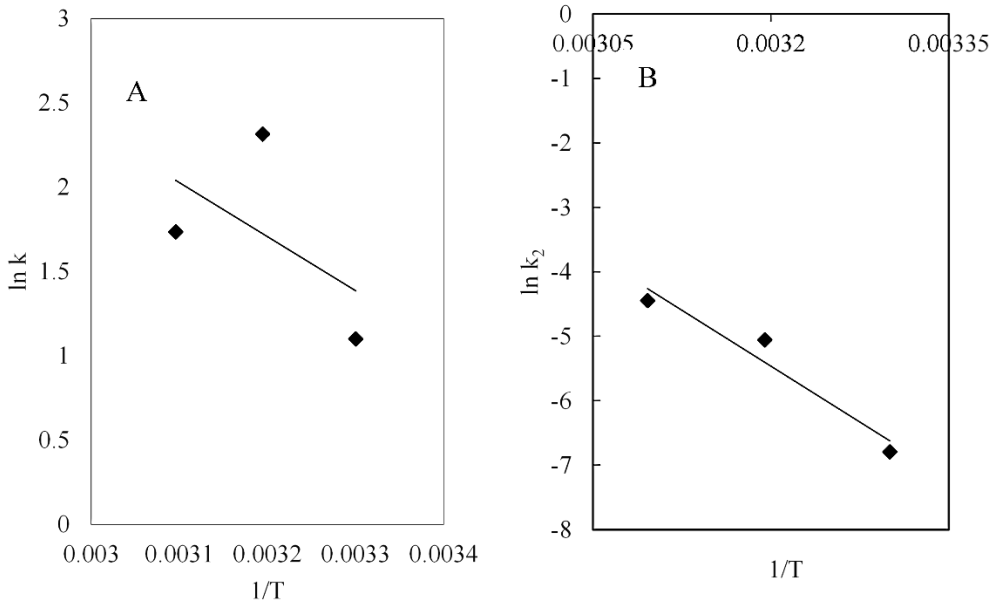

Figure S11. Thermodynamic study for (A) Plot of ln k versus 1/T for estimation of thermodynamic parameters and (B) plot of ln k2 versus 1/T for estimation of E_a for the adsorption of FeAlCr (1)

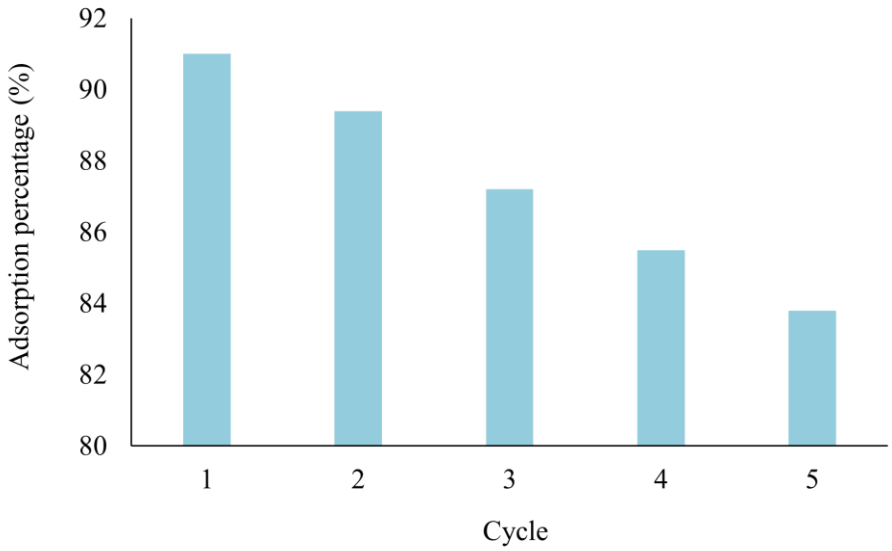

Figure S12. Reusability of FeAl adsorbent over five consecutive Cr ions adsorption–desorption cycles under optimal conditions of 0.05 g dosage, initial concentration of 25 mg L^{-1}, pH 7 and temperature of 40 °C.
